# Supplementary material for: eModel-BDB: a database of comparative structure models of drug-target interactions from the Binding Database
Source: Gigascience. 2018 Jul 24;7(8):giy091. doi: 10.1093/gigascience/giy091 (PMC6131211; doi:10.1093/gigascience/giy091)

GigaScience

eModel-BDB: A database of comparative structure models of drug-target interactions from the Binding Database

--Manuscript Draft--

**Manuscript Number:**	GIGA-D-17-00258
**Full Title:**	eModel-BDB: A database of comparative structure models of drug-target interactions from the Binding Database
**Article Type:**	Data Note
**Funding Information:**	National Institute of General Medical Sciences (R35GM119524) — Dr. Michal Brylinski
**Abstract:**	Background<br>The structural information on proteins in their ligand-bound conformational state is invaluable for protein function studies and rational drug design. Compared to the number of available sequences, the repertoire of the experimentally determined structures of holo-proteins is not only limited, but also these structures do not always include pharmacologically relevant compounds at their binding sites. In addition, binding affinity databases provide vast quantities of information on interactions between drug-like molecules and their targets, however, often lacking structural data. On that account, there is a need for computational methods to complement existing repositories by constructing the atomic-level models of drug-protein assemblies that will not be determined experimentally in the near future.<br>Findings<br>We created eModel-BDB, a database of 200,008 high-quality, comparative models of drug-bound proteins based on interaction data obtained from the Binding Database. Complex models in eModel-BDB were generated with a collection of the state-of-the-art techniques, including meta-threading, template-based structure modeling, refinement and binding site detection, and similarity-based docking. In addition to a rigorous quality control maintained during dataset generation, a subset of weakly homologous models were selected for the retrospective validation against experimental structural data recently deposited to the Protein Data Bank. Validation results indicate that eModel-BDB contains high-quality models not only at the global protein structure level, but also with respect to the atomic details of the bound ligands.<br>Conclusions<br>Freely available eModel-BDB can be used to support structure-based drug discovery and repositioning, drug target identification, and protein structure determination.
**Corresponding Author:**	Michal Brylinski, Ph.D.<br>Louisiana State University<br>Baton Rouge, Louisiana UNITED STATES
**Corresponding Author Secondary Information:**	
**Corresponding Author's Institution:**	Louisiana State University
**Corresponding Author's Secondary Institution:**	
**First Author:**	Michal Brylinski, Ph.D.
**First Author Secondary Information:**	
**Order of Authors:**	Michal Brylinski, Ph.D.<br>Misagh Naderi
**Order of Authors Secondary Information:**	
**Opposed Reviewers:**	Jeffrey Skolnick<br>Georgia Institute of Technology<br><br>Conflict of interests

Powered by Editorial Manager® and ProduXion Manager® from Aries Systems Corporation

Additional Information:	
**Question**	**Response**
Are you submitting this manuscript to a special series or article collection?	No
**Experimental design and statistics**<br><br>Full details of the experimental design and statistical methods used should be given in the Methods section, as detailed in our Minimum Standards Reporting Checklist. Information essential to interpreting the data presented should be made available in the figure legends.<br><br>Have you included all the information requested in your manuscript?	Yes
**Resources**<br><br>A description of all resources used, including antibodies, cell lines, animals and software tools, with enough information to allow them to be uniquely identified, should be included in the Methods section. Authors are strongly encouraged to cite Research Resource Identifiers (RRIDs) for antibodies, model organisms and tools, where possible.<br><br>Have you included the information requested as detailed in our Minimum Standards Reporting Checklist?	Yes
**Availability of data and materials**<br><br>All datasets and code on which the conclusions of the paper rely must be either included in your submission or deposited in publicly available repositories (where available and ethically appropriate), referencing such data using a unique identifier in the references and in the “Availability of Data and Materials” section of your manuscript.<br><br>Have you have met the above requirement as detailed in our Minimum Standards Reporting Checklist?	Yes

Manuscript Click here to download Manuscript Manuscript.docx

*e*Model-BDB: A database of comparative structure models of drug-target interactions from the Binding Database

by

Misagh Naderi[1] and Michal Brylinski[1,2*]

[1]Department of Biological Sciences, Louisiana State University, Baton Rouge, LA 70803, USA

[2]Center for Computation & Technology, Louisiana State University, Baton Rouge, LA 70803, USA

[*] Corresponding author: Michal Brylinski

Email addresses: mnader5@lsu.edu (Misagh Naderi)

michal@brylinski.org (Michal Brylinski)

Phone: (225) 578-2791

Fax: (225) 578-2597

Abstract

Background

The structural information on proteins in their ligand-bound conformational state is invaluable for protein function studies and rational drug design. Compared to the number of available sequences, the repertoire of the experimentally determined structures of holo-proteins is not only limited, but also these structures do not always include pharmacologically relevant compounds at their binding sites. In addition, binding affinity databases provide vast quantities of information on interactions between drug-like molecules and their targets, however, often lacking structural data. On that account, there is a need for computational methods to complement existing repositories by constructing the atomic-level models of drug-protein assemblies that will not be determined experimentally in the near future.

Findings

We created *e*Model-BDB, a database of 200,008 high-quality, comparative models of drug-bound proteins based on interaction data obtained from the Binding Database. Complex models in *e*Model-BDB were generated with a collection of the state-of-the-art techniques, including meta-threading, template-based structure modeling, refinement and binding site detection, and similarity-based docking. In addition to a rigorous quality control maintained during dataset generation, a subset of weakly homologous models were selected for the retrospective validation against experimental structural data recently deposited to the Protein Data Bank. Validation results indicate that *e*Model-BDB contains high-quality models not only at the global protein structure level, but also with respect to the atomic details of the bound ligands.

Conclusions

Freely available *e*Model-BDB can be used to support structure-based drug discovery and repositioning, drug target identification, and protein structure determination.

Keywords: *e*Model-BDB, *e*Thread, *e*FindSite, BindingDB, homology modeling, comparative modeling, binding pocket prediction, similarity-based docking, protein function, drug targets

Data Description

Context

Structural bioinformatics is becoming an increasingly important component of modern drug discovery. Despite significant advances in experimental methods to acquire protein structures, such as X-ray crystallography and nuclear magnetic resonance, technical limitations and expensive procedures make it unlikely to have the experimental structures of all known protein sequences in the near future. For example, as of October 2017, the number of gene products in the Reference Sequence Database [1] is 9.5×10^7. In contrast, the number of experimentally determined protein structures in the Protein Data Bank (PDB) [2] is 130,750, which reduces to 46,893 structures after removing similar proteins at 95% sequence identity. Genome sequencing currently produces as many as 13 million protein sequences each year, whereas only an average number of 8,872 protein structures are solved experimentally at the same time. Since this disparity between the number of available sequences and structures will likely continue to grow, a high-throughput computational modeling is expected to play a significant role in biomedical sciences by generating 3D models for those proteins whose structures will not be determined in the near future.

In addition to protein sequence and structure repositories, the Binding Database (BindingDB) provides comprehensive information on interactions between small, drug-like molecules and proteins considered to be drug targets collected from affinity measurements [3]. The BindingDB can be used to identify protein targets for small molecules and bioactive compounds for new proteins, as well as to conduct virtual screening with ligand-based methods. As of October 2017, BindingDB contains 1,391,403 binding data, however, only 2,291 ligand-protein crystal structures with BindingDB affinity measurements are available in the PDB. To bridge this gap, we created *e*Model-BDB, a new database of 200,008 high-quality drug-protein complex models involving 108,363 unique drug-like compounds and 2,791 proteins from the BindingDB. This repository was constructed with a state-of-the-art protocol to generate protein models in their ligand-bound conformational state, employing meta-threading, pocket detection, and protein structure and ligand chemical alignment techniques.

*e*Model-BDB roughly quadruples the current structural information on known drug-protein complexes.

To fully appreciate the immensity of structural data included in *e*Model-BDB, we estimate the time required to solve an equal number of drug-protein assemblies. Figure 1 shows that at the current pace, 2,447 ligand-bound protein structures containing 607 non-redundant complexes are deposited to the PDB each month. Therefore, it would take about 329 months for 200,008 unique complex structures to be determined experimentally. In contrast to other databases comprising protein models in the unbound conformational state generated through traditional structure modeling [4, 5], *e*Model-BDB includes annotated structure models of drug-protein complexes with known binding affinities. It provides high-quality data to support structure-based drug discovery as well as repurposing of known drugs based on binding pocket and ligand similarities. In addition, the information provided by *e*Model-BDB can be utilized to facilitate experimental structure determination by developing protocols to stabilize proteins with ligands. The protocol to construct *e*Model-BDB described in this communication is based entirely on open source software to ensure that any researcher is able to produce new holo-protein models as more data becomes available in the PDB and BindingDB.

Methods

Drug-bound protein complexes in *e*Model-BDB are generated with a template-based approach. The first phase is to construct structure models for protein sequences 50-999 amino acids in length obtained from BindingDB with *e*Thread [6], which supports both close as well as remote homology modeling. *e*Thread employs Modeller, a commonly used comparative modeling program [7], to build apo-protein structures based on alignments produced by three fold recognition algorithms, HH-suite [8], SparksX [9], and RaptorX [10]. Subsequently, side-chain positions and hydrogen-bonding networks in the initial models are improved with ModRefiner, a program to refine protein structures at the atomic-level with a composite physics- and knowledge-based force field [11]. The quality assessment of refined models is carried out with ModelEvaluator [12] in terms of the estimated Global Distance Test score (GDT-score). Out of 5,501 models of BindingDB proteins, 4,906 were assigned an estimated GDT-score of ≥0.4.

Confident structure models are further annotated with binding pockets and residues by *e*FindSite [13], which also computes a calibrated pocket confidence score. *e*FindSite detected 2,922 high-, 644 moderate-, and 776 low-confidence pockets in the *e*Thread models of BindingDB targets. At this point, BindingDB drugs can be assigned to the predicted pockets with fingerprint-based virtual screening [14]. Specifically, for a given drug-target pair in the BindingDB, we compute a rank of the drug against pockets detected by *e*FindSite, where the remaining BindingDB compounds are used as the background library. Top one, two and three pockets are considered for high-, moderate- and low-confidence targets, respectively. A drug matches the predicted pocket if it is ranked within the top 20% of the screening library. With this protocol, we matched 108,363 drugs to binding pockets identified in their target proteins.

In the next phase, drug molecules are positioned within the predicted pockets with a two-step, similarity-based docking protocol. First, globally similar ligand-bound templates from the PDB, identified by *e*FindSite to have a similar pocket as the BindingDB protein, are superimposed onto the apo-model. Proteins are aligned with Fr-TM-align [15] employing the Template Modeling score (TM-score) [16] to measure the global structure similarity. Subsequently, the BindingDB compound is aligned onto the template-bound ligand in order to place it in the predicted pocket of the apo-model. Here, we use chemical alignments constructed with kcombu [17], which also reports the chemical similarity between the BindingDB compound and the template-bound ligand measured by the Tanimoto coefficient (TC). Since a perfect case corresponds to both a TM-score and a TC of 1.0, we introduce a new metric, the Perfect Match Distance (PMD), combining protein structure and ligand chemical similarity values:

$$PMD = \sqrt{(1 - TM\text{-}score)^2 + (1 - TC)^2} \quad \text{Eq. 1.}$$

PMD is simply the Cartesian distance from the perfect match on the TM-score/TC plane. In order to generate only high-quality holo-models, those cases with a PMD of >0.6 are excluded from the modeling process. This PMD cutoff was chosen to ensure that both TM-score and TC for the selected templates are always above their individual significance threshold

values of $\geq$0.4 [16, 17]. Further, for those cases having multiple ligand-bound templates satisfying the PMD criterion of $\leq$0.6, a template with the shortest PMD is selected to build the holo-model of the BindingDB complex. Encouragingly, the median TM-score and TC for ligand-bound templates used to build *e*Model-BDB are as high as 0.81 and 0.67, respectively. Previous studies show that the probability for a protein pair to belong to the same fold is 98% when the TM-score is close to 0.8 [18]. In addition, it was demonstrated that the root-mean-square deviation (RMSD) over ligand non-hydrogen atoms for similarity-based docking conducted with the TC in the range of 0.6-0.8 is typically 2-3 Å [19].

In the final phase, protein models are rebuilt in the presence of the docked BindingDB compounds with Modeller. To make sure that the binding site is remodeled to accommodate the specific ligand, binding residues identified by *e*FindSite are removed from the initial model while enforcing the presence of secondary structure predicted by PSIPRED [20]. The resulting models are further annotated with the ligand-protein interaction score according to the Distance-scaled Finite Ideal-gas REference (DFIRE) potential. The *e*Model-BDB database contains atomic-level structure models of 200,008 drug-protein interactions from BindingDB, comprising 2,791 non-redundant proteins and 108,363 drug-like compounds.

Data validation and quality control

The quality control is pertinent to both protein structure modeling as well as binding site prediction. The quality of protein models is assessed with ModelEvaluator employing various structural features to compute the absolute quantitative score with a support vector regression. This approach assigns the GDT-score to a model by analyzing its secondary structure, relative solvent accessibility, contact map, and β-sheet structure. It has been demonstrated that GDT-scores estimated by ModelEvaluator for template-based models are highly correlated with the actual values with the Pearson correlation coefficient of 0.82 [12]. The *e*Model-BDB database comprises protein models whose median GDT-score is 0.74, therefore, we expect that the vast majority of these structures are highly accurate. Further, the median confidence of binding sites predicted by *e*FindSite to match BindingDB ligands is 0.93. We showed previously that confidence scores of >0.8 assigned by *e*FindSite correspond to the

Mathews correlation coefficient (MCC) of ≥0.6 for predicted binding residues [13]. On that account, we expect that the majority of binding sites for BindingDB drugs are annotated with a high accuracy as well. Note that in contrast to other pocket predictors, *e*FindSite annotations and confidence assignments are to some extent independent on the quality protein models.

In addition to the rigorous quality control maintained during dataset generation, *e*Model-BDB is validated retrospectively against experimental structures recently deposited to the PDB. The construction of the structure models of BindingDB interactions has been completed in January 2017, therefore, we selected 7,012 experimental structures deposited to the PDB after February 2017 to validate *e*Model-BDB structures. Further, the validation protocol is made more challenging by including only those models built from remote homology at a template-target sequence identity of <40%. In order to maximize the validation coverage, we use the recently determined structures of *e*Model-BDB targets and their homologs with at least 40% sequence identity. The first violin in Figure 2 shows that the median TM-score of *e*Model-BDB vs. experimental structures is as high as 0.90 with as many as 99.7% of the models having a TM-score of ≥0.4.

The accuracy of pocket prediction is validated by first superposing the experimental holo structure onto the *e*Model-BDB model and then calculating the distance between the geometric center of a bound ligand in the experimental complex and the pocket center predicted by *e*FindSite in the model. The second violin in Figure 2 shows that the median pocket distance is only 5.5 Å with 59.6% of pockets predicted within 6 Å. Finally, we calculate the RMSD over non-hydrogen atoms between the BindingDB drug in the *e*Model-BDB structure and the bound ligand in the superposed experimental complex. The last violin in Figure 2 shows that the median RMSD is 2.9 Å and it is ≤3 Å for 52.7% of BindingDB compounds. Overall, the quality assessment as well as the independently obtained validation results demonstrate that the *e*Model-BDB database contains high-quality models closely resembling experimentally determined structures, not only at the global structure level, but also at the level of binding pockets and bound ligands.

Re-use potential

*e*Model-BDB is generated to support rational drug development projects. These data can directly aid structure-based drug discovery pipelines and protein function analysis by providing atomic-level models of a large set of drug-protein interactions with known affinities. An important application of *e*Model-BDB is computational drug repositioning, i.e. finding new indications for existing drugs [21]. Although drug repurposing holds a significant promise to speed up drug development, particularly for diseases considered to be unprofitable, its major bottleneck is the scarce structural information on druggable pockets. On that account, a diverse dataset of drug-like small molecules bound to high-quality models with accurately annotated pockets provide an invaluable resource for drug repositioning. It is noteworthy that computational drug repurposing employing drug-bound protein models and sequence order-independent pocket matching algorithms [22, 23], has recently revealed new opportunities to combat rare diseases [24, 25].

Binding sites in *e*Model-BDB can also be matched to pockets predicted in potential drug targets in order to determine whether these proteins are druggable or not. If a new pocket aligns well with drug-bound pockets in *e*Model-BDB then it is likely going to be druggable. That being the case, our data can be utilized right at the outset of drug discovery, in the target identification phase. Finally, ligand binding can significantly help stabilize a protein, particularly from the point of view of the conformational stability [26]. *e*Model-BDB can, therefore, inform crystallography efforts by suggesting possible compounds binding to certain protein targets at either the active or allosteric sites in order to increase the chances of successful crystallization.

Availability of supporting data

*e*Model-BDB data will be made freely available through the GigaScience repository should this manuscript be accepted for publication.

Declarations

List of abbreviations

BindingDB, Binding Database; DFIRE, Distance-scaled Finite Ideal-gas REference; GDT-score, Global Distance Test score; MCC, Mathews correlation coefficient; PMD, Perfect Match Distance; PDB, Protein Data Bank; RMSD, root-mean-square deviation; TC, Tanimoto coefficient; TM-score, Template Modeling score

Competing interests

The authors declare that they have no competing interests.

Funding

Research reported in this publication was supported by the National Institute of General Medical Sciences of the National Institutes of Health under Award Number R35GM119524.

Authors' contributions

MB prepared protein models, annotated binding pockets, and validated protein structures and pockets. MN constructed, refined, and validated drug-bound models. MN and MB wrote the paper.

Acknowledgements

Authors are grateful to Louisiana State University for providing computing resources.

References

1. O'Leary NA, Wright MW, Brister JR, Ciufo S, Haddad D, McVeigh R, et al. Reference sequence (RefSeq) database at NCBI: current status, taxonomic expansion, and functional annotation. Nucleic Acids Res. 2016;44 D1:D733-45. doi:10.1093/nar/gkv1189.
2. Berman HM, Westbrook J, Feng Z, Gilliland G, Bhat TN, Weissig H, et al. The Protein Data Bank. Nucleic Acids Res. 2000;28 1:235-42.
3. Liu T, Lin Y, Wen X, Jorissen RN and Gilson MK. BindingDB: a web-accessible database of experimentally determined protein-ligand binding affinities. Nucleic Acids Res. 2007;35 Database issue:D198-201. doi:10.1093/nar/gkl999.
4. Castrignano T, De Meo PD, Cozzetto D, Talamo IG and Tramontano A. The PMDB Protein Model Database. Nucleic Acids Res. 2006;34 Database issue:D306-9. doi:10.1093/nar/gkj105.

5. Sanchez R, Pieper U, Mirkovic N, de Bakker PI, Wittenstein E and Sali A. MODBASE, a database of annotated comparative protein structure models. Nucleic Acids Res. 2000;28 1:250-3.
6. Brylinski M and Lingam D. eThread: a highly optimized machine learning-based approach to meta-threading and the modeling of protein tertiary structures. PLoS One. 2012;7 11:e50200. doi:10.1371/journal.pone.0050200.
7. Sali A and Blundell TL. Comparative protein modelling by satisfaction of spatial restraints. J Mol Biol. 1993;234 3:779-815. doi:10.1006/jmbi.1993.1626.
8. Remmert M, Biegert A, Hauser A and Soding J. HHblits: lightning-fast iterative protein sequence searching by HMM-HMM alignment. Nat Methods. 2011;9 2:173-5. doi:10.1038/nmeth.1818.
9. Yang Y, Faraggi E, Zhao H and Zhou Y. Improving protein fold recognition and template-based modeling by employing probabilistic-based matching between predicted one-dimensional structural properties of query and corresponding native properties of templates. Bioinformatics. 2011;27 15:2076-82. doi:10.1093/bioinformatics/btr350.
10. Ma J, Wang S, Zhao F and Xu J. Protein threading using context-specific alignment potential. Bioinformatics. 2013;29 13:i257-65. doi:10.1093/bioinformatics/btt210.
11. Xu D and Zhang Y. Improving the physical realism and structural accuracy of protein models by a two-step atomic-level energy minimization. Biophys J. 2011;101 10:2525-34. doi:10.1016/j.bpj.2011.10.024.
12. Wang Z, Tegge AN and Cheng J. Evaluating the absolute quality of a single protein model using structural features and support vector machines. Proteins. 2009;75 3:638-47. doi:10.1002/prot.22275.
13. Brylinski M and Feinstein WP. eFindSite: improved prediction of ligand binding sites in protein models using meta-threading, machine learning and auxiliary ligands. J Comput Aided Mol Des. 2013;27 6:551-67. doi:10.1007/s10822-013-9663-5.
14. Feinstein WP and Brylinski M. eFindSite: Enhanced fingerprint-based virtual screening against predicted ligand binding sites in protein models. Mol Inform. 2014;33 2:135-50. doi:10.1002/minf.201300143.
15. Pandit SB and Skolnick J. Fr-TM-align: a new protein structural alignment method based on fragment alignments and the TM-score. BMC Bioinformatics. 2008;9:531. doi:10.1186/1471-2105-9-531.
16. Zhang Y and Skolnick J. Scoring function for automated assessment of protein structure template quality. Proteins. 2004;57 4:702-10. doi:10.1002/prot.20264.
17. Kawabata T. Build-up algorithm for atomic correspondence between chemical structures. Journal of chemical information and modeling. 2011;51 8:1775-87. doi:10.1021/ci2001023.
18. Xu J and Zhang Y. How significant is a protein structure similarity with TM-score = 0.5? Bioinformatics. 2010;26 7:889-95. doi:10.1093/bioinformatics/btq066.
19. Brylinski M. Nonlinear scoring functions for similarity-based ligand docking and binding affinity prediction. Journal of chemical information and modeling. 2013;53 11:3097-112. doi:10.1021/ci400510e.
20. Jones DT. Protein secondary structure prediction based on position-specific scoring matrices. J Mol Biol. 1999;292 2:195-202. doi:10.1006/jmbi.1999.3091.

21. Haupt VJ and Schroeder M. Old friends in new guise: repositioning of known drugs with structural bioinformatics. Brief Bioinform. 2011;12 4:312-26. doi:10.1093/bib/bbr011.
22. Brylinski M. eMatchSite: sequence order-independent structure alignments of ligand binding pockets in protein models. PLoS Comput Biol. 2014;10 9:e1003829. doi:10.1371/journal.pcbi.1003829.
23. Brylinski M. Local alignment of ligand binding sites in proteins for polypharmacology and drug repositioning. Methods Mol Biol. 2017;1611:109-22. doi:10.1007/978-1-4939-7015-5_9.
24. Naderi M, Lemoine JM and Brylinski M. Large-scale computational drug repositioning to find treatments for rare diseases. submitted.
25. Brylinski M, Naderi M and Lemoine JM. eRepo-ORP: Exploring the opportunity space to combat orphan diseases with existing drugs. submitted.
26. Deller MC, Kong L and Rupp B. Protein stability: a crystallographer's perspective. Acta Crystallogr F Struct Biol Commun. 2016;72 Pt 2:72-95. doi:10.1107/S2053230X15024619.

Figure captions

Figure 1. Deposition rate of ligand-bound structures to the Protein Data Bank. At any given time, we counted the total number of protein chains binding small molecules (light gray squares and a dashed line) and the number of unique complex structures obtained by clustering individual chains at 80% sequence identity (dark gray circles and a solid line). N_t and N_u in the linear regression equations are the total and unique number of ligand-protein complexes, respectively, and *m* stands for month.

Figure 2. Violin and box plots for the distribution of validation scores. The validation is conducted for remote homology protein models constructed by January 2017 against the experimental structures of either target proteins or their close homologs deposited to the PDB after February 2017. TM-score measures the global structure similarity. The pocket distance and the ligand RMSD are calculated upon the superposition of the experimental structure onto the model. Pocket distance is measured between the geometric center of the ligand in the experimental structure and the predicted pocket center, whereas the RMSD is calculated over non-hydrogen atoms according to the chemical alignment reported by kcombu.

Figure 1

Click here to download Figure Figure1.tif

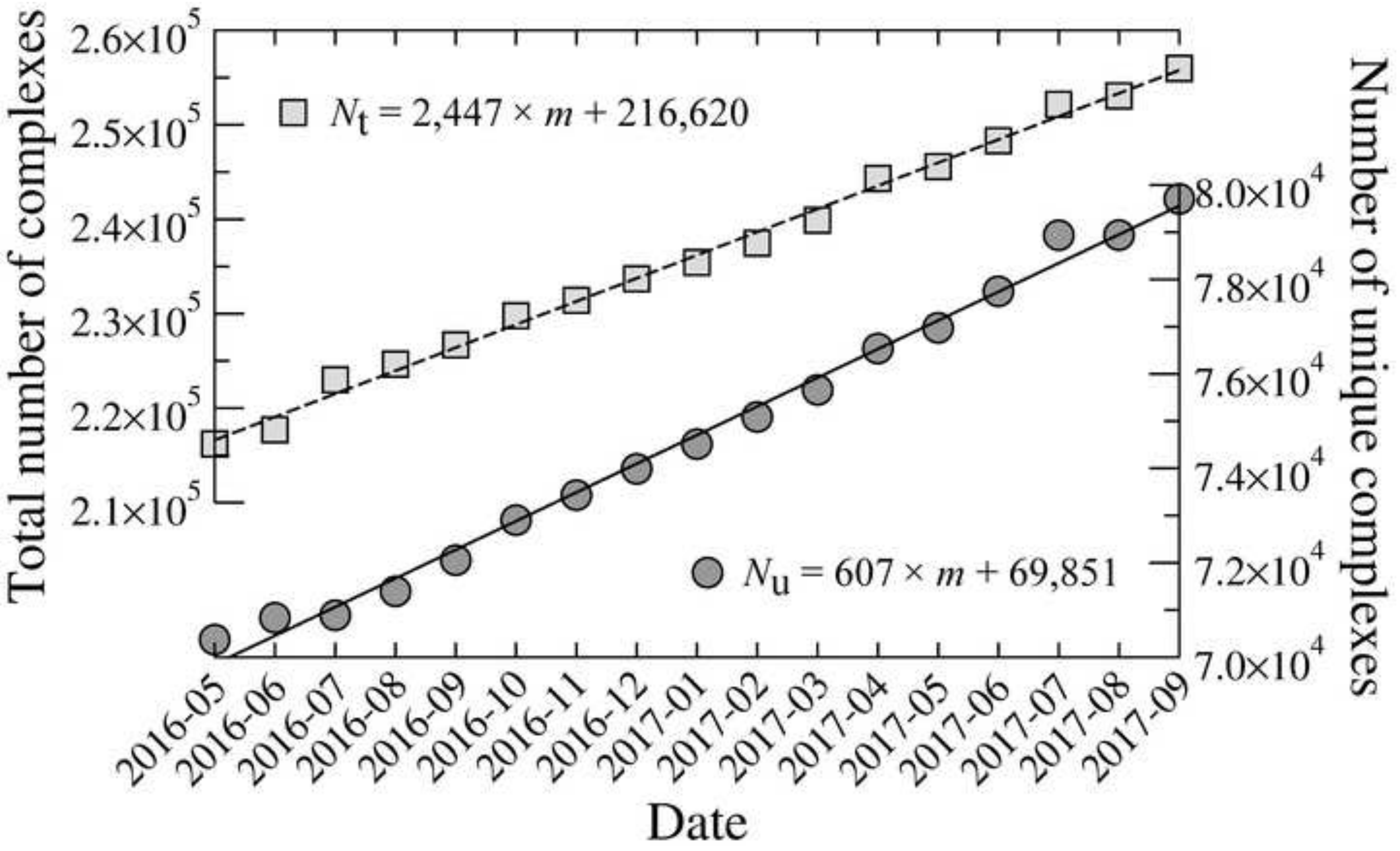

Click here to download Figure Figure2.tif

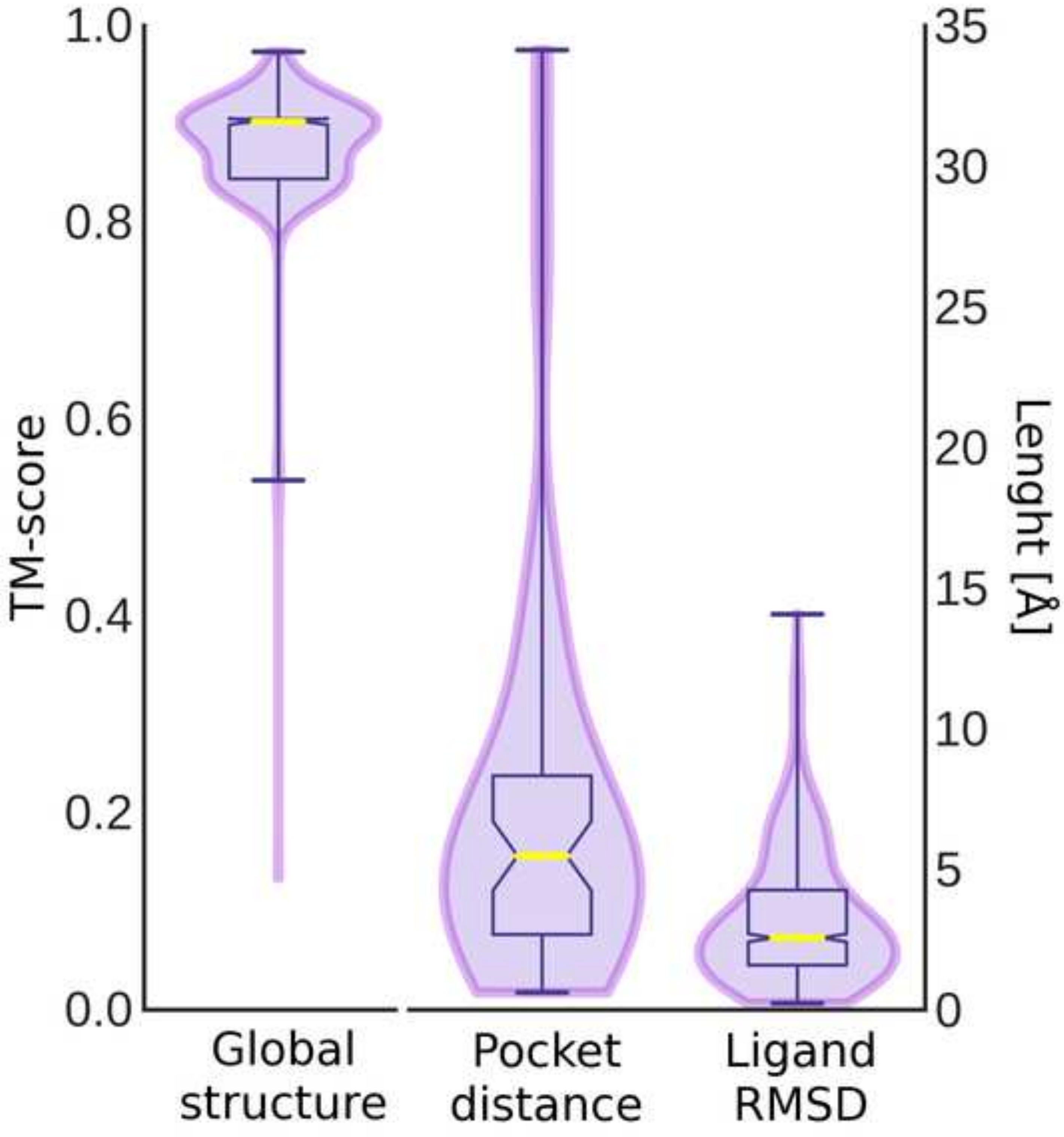

Supplement: GIGA-D-17-00258_Original_Submission.pdf [file giy091_giga-d-17-00258_original_submission.pdf]
