# Supplementary material for: eModel-BDB: a database of comparative structure models of drug-target interactions from the Binding Database
Source: Gigascience. 2018 Jul 24;7(8):giy091. doi: 10.1093/gigascience/giy091 (PMC6131211; doi:10.1093/gigascience/giy091)
Supplement: GIGA-D-17-00258_Revision_2.pdf [file giy091_giga-d-17-00258_revision_2.pdf]

GigaScience

eModel-BDB: A database of comparative structure models of drug-target interactions from the Binding Database

--Manuscript Draft--

**Manuscript Number:**	GIGA-D-17-00258R2
**Full Title:**	eModel-BDB: A database of comparative structure models of drug-target interactions from the Binding Database
**Article Type:**	Research
**Funding Information:**	National Institute of General Medical Sciences (R35GM119524) — Dr. Michal Brylinski
**Abstract:**	Background. The structural information on proteins in their ligand-bound conformational state is invaluable for protein function studies and rational drug design. Compared to the number of available sequences, the repertoire of the experimentally determined structures of holo-proteins is not only limited, but also these structures do not always include pharmacologically relevant compounds at their binding sites. In addition, binding affinity databases provide vast quantities of information on interactions between drug-like molecules and their targets, however, often lacking structural data. On that account, there is a need for computational methods to complement existing repositories by constructing the atomic-level models of drug-protein assemblies that will not be determined experimentally in the near future. Results. We created eModel-BDB, a database of 200,005 comparative models of drug-bound proteins based on interaction data obtained from the Binding Database. Complex models in eModel-BDB were generated with a collection of the state-of-the-art techniques, including protein meta-threading, template-based structure modeling, refinement and binding site detection, and ligand similarity-based docking. In addition to a rigorous quality control maintained during dataset generation, a subset of weakly homologous models were selected for the retrospective validation against experimental structural data recently deposited to the Protein Data Bank. Validation results indicate that eModel-BDB contains models that are accurate not only at the global protein structure level, but also with respect to the atomic details of bound ligands. Conclusions. Freely available eModel-BDB can be used to support structure-based drug discovery and repositioning, drug target identification, and protein structure determination.
**Corresponding Author:**	Michal Brylinski, Ph.D.<br>Louisiana State University<br>Baton Rouge, Louisiana UNITED STATES
**Corresponding Author Secondary Information:**	
**Corresponding Author's Institution:**	Louisiana State University
**Corresponding Author's Secondary Institution:**	
**First Author:**	Misagh Naderi
**First Author Secondary Information:**	
**Order of Authors:**	Misagh Naderi
	Rajiv Gandhi Govindaraj
	Michal Brylinski, Ph.D.
**Order of Authors Secondary Information:**	
**Response to Reviewers:**	Our responses are provided in a separate document "ResponseLetter.pdf" uploaded as Supplementary Material.
**Additional Information:**	

Powered by Editorial Manager® and ProduXion Manager® from Aries Systems Corporation

Question	Response
Are you submitting this manuscript to a special series or article collection?	No
**Experimental design and statistics**<br><br>Full details of the experimental design and statistical methods used should be given in the Methods section, as detailed in our Minimum Standards Reporting Checklist. Information essential to interpreting the data presented should be made available in the figure legends.<br><br>Have you included all the information requested in your manuscript?	Yes
**Resources**<br><br>A description of all resources used, including antibodies, cell lines, animals and software tools, with enough information to allow them to be uniquely identified, should be included in the Methods section. Authors are strongly encouraged to cite Research Resource Identifiers (RRIDs) for antibodies, model organisms and tools, where possible.<br><br>Have you included the information requested as detailed in our Minimum Standards Reporting Checklist?	Yes
**Availability of data and materials**<br><br>All datasets and code on which the conclusions of the paper rely must be either included in your submission or deposited in publicly available repositories (where available and ethically appropriate), referencing such data using a unique identifier in the references and in the "Availability of Data and Materials" section of your manuscript.	Yes

Have you have met the above requirement as detailed in our Minimum Standards Reporting Checklist?	

Powered by Editorial Manager® and ProduXion Manager® from Aries Systems Corporation

Manuscript Click here to access/download;Manuscript;eModelBDB.docx
Click here to view linked References

*e*Model-BDB: A database of comparative structure models of drug-target interactions from the Binding Database

by

Misagh Naderi[1], Rajiv Gandhi Govindaraj[1] and Michal Brylinski[1,2*]

[1]Department of Biological Sciences, Louisiana State University, Baton Rouge, LA 70803, USA

[2]Center for Computation & Technology, Louisiana State University, Baton Rouge, LA 70803, USA

[*] Corresponding author: Michal Brylinski

Email addresses: mnader5@lsu.edu (Misagh Naderi), ORCID: 0000-0001-9866-5366

rgovinda@lsu.edu (Rajiv Gandhi Govindaraj), ORCID:0000-0003-2364-5429

michal@brylinski.org (Michal Brylinski), ORCID: 0000-0002-6204-2869

Phone: (225) 578-2791

Fax: (225) 578-2597

Abstract

***Background*.** The structural information on proteins in their ligand-bound conformational state is invaluable for protein function studies and rational drug design. Compared to the number of available sequences, the repertoire of the experimentally determined structures of holo-proteins is not only limited, but also these structures do not always include pharmacologically relevant compounds at their binding sites. In addition, binding affinity databases provide vast quantities of information on interactions between drug-like molecules and their targets, however, often lacking structural data. On that account, there is a need for computational methods to complement existing repositories by constructing the atomic-level models of drug-protein assemblies that will not be determined experimentally in the near future.

***Results*.** We created *e*Model-BDB, a database of 200,005 comparative models of drug-bound proteins based on 1,391,403 interaction data obtained from the Binding Database and the PDB library of January 31, 2017. Complex models in *e*Model-BDB were generated with a collection of the state-of-the-art techniques, including protein meta-threading, template-based structure modeling, refinement and binding site detection, and ligand similarity-based docking. In addition to a rigorous quality control maintained during dataset generation, a subset of weakly homologous models were selected for the retrospective validation against experimental structural data recently deposited to the Protein Data Bank. Validation results indicate that *e*Model-BDB contains models that are accurate not only at the global protein structure level, but also with respect to the atomic details of bound ligands.

***Conclusions*.** Freely available *e*Model-BDB can be used to support structure-based drug discovery and repositioning, drug target identification, and protein structure determination.

Keywords: *e*Model-BDB, *e*Thread, *e*FindSite, BindingDB, homology modeling, comparative modeling, binding pocket prediction, similarity-based docking, protein function, drug targets

Background

Structural bioinformatics is becoming an increasingly important component of modern drug discovery. Despite significant advances in experimental methods to acquire protein structures, such as X-ray crystallography, nuclear magnetic resonance, and cryo-electron microscopy, technical limitations and expensive procedures make it unlikely to have the experimental structures of all known protein sequences in the near future. For example, more than 110 million gene products are included in the Reference Sequence Database [1] as of June 2018. In contrast, the number of experimentally determined protein structures in the Protein Data Bank (PDB) [2] is 140,824, which reduces to 51,990 structures after removing similar proteins at 95% sequence identity. Genome sequencing currently produces as many as 13 million protein sequences each year, whereas only 8,872 protein structures are solved experimentally at the same time on average. Since this disparity between the number of available sequences and structures will likely continue to grow, high-throughput computational modeling is expected to play a significant role in biomedical sciences by generating 3D models for those proteins whose structures will not be determined in the near future.

In addition to protein sequence and structure repositories, the Binding Database (BindingDB) provides comprehensive information on interactions between small, drug-like molecules and proteins considered to be drug targets collected from affinity measurements [3]. The BindingDB can be used to identify protein targets for small molecules and bioactive compounds for new proteins, as well as to conduct virtual screening with ligand-based methods. As of June 2018, BindingDB contains 1,450,120 binding data, however, only 2,291 ligand-protein crystal structures with BindingDB affinity measurements are available in the PDB. To bridge this gap, we created *e*Model-BDB, a new database of 200,005 high-quality drug-protein complex models involving 108,363 unique drug-like compounds and 2,791 proteins from the BindingDB. This repository was constructed with a state-of-the-art protocol to generate protein models in their ligand-bound conformational state, employing meta-threading, pocket detection, and protein structure and ligand chemical alignment techniques. *e*Model-BDB significantly expands the current structural information on known drug-protein complexes.

To fully appreciate the immensity of the structural data included in *e*Model-BDB, we estimate the time required to solve an equal number of drug-protein assemblies. Figure 1 shows that at the current pace, 2,447 ligand-bound protein structures containing 607 non-redundant complexes are deposited to the PDB each month. Therefore, it would take about 329 months for 200,005 unique complex structures to be determined experimentally. In contrast to other databases comprising protein models in the unbound conformational state generated through traditional structure modeling [4, 5], *e*Model-BDB includes annotated structure models of drug-protein complexes with known binding affinities. It provides high-quality data to support structure-based drug discovery as well as repurposing of known drugs based on binding pocket and ligand similarities. In addition, the information provided by *e*Model-BDB can be utilized to facilitate experimental structure determination by developing protocols to stabilize proteins with ligands. The protocol to construct *e*Model-BDB described in this communication is based entirely on open source software to ensure that any researcher is able to produce new holo-protein models as more data becomes available in the PDB and BindingDB.

Methods

Protein structure modeling

Drug-bound protein complexes in *e*Model-BDB are generated with a template-based approach. The first phase is to construct structure models for single protein chains 50-999 amino acids in length obtained from BindingDB with *e*Thread [6], which supports both close and remote homology modeling. *e*Thread employs Modeller, a commonly used comparative modeling program (RRID:SCR_008395)[7], to build apo-protein structures based on alignments produced by three fold recognition algorithms, HH-suite [8], SparksX [9], and RaptorX [10]. Subsequently, side-chain positions and hydrogen-bonding networks in the initial models are improved with ModRefiner, a program to refine protein structures at the atomic-level with a composite physics- and knowledge-based force field [11]. The quality assessment of refined models is carried out with ModelEvaluator [12] in terms of the estimated Global Distance Test score (GDT-score). Out of 5,501 BindingDB proteins, 4,906 were assigned an estimated GDT-score of ≥0.4 indicating good quality models [13, 14].

Ligand-binding site identification

Confident structure models with a GDT-score of ≥0.4 are further annotated with binding pockets and residues by *e*FindSite [15], which also computes a calibrated pocket confidence score. *e*FindSite detected 2,922 high-, 644 moderate-, and 776 low-confidence pockets in the *e*Thread models of BindingDB targets. At this point, BindingDB drugs can be assigned to the predicted pockets with fingerprint-based virtual screening. Specifically, for a given drug-target pair in the BindingDB, we compute a rank of the drug against pockets detected by *e*FindSite, where the remaining BindingDB compounds are used as the background library. *e*FindSite conducts virtual screening with a set of molecular fingerprints and physicochemical properties calculated for ligands extracted from weakly homologous template structures [16]. Top one, two and three pockets are considered for high-, moderate- and low-confidence targets, respectively. A drug matches the predicted pocket if it is ranked within the top 20% of the screening library. With this protocol, we matched 108,363 drugs to binding pockets identified in their target proteins.

Similarity-based ligand docking

In the next phase, drug molecules are positioned within the predicted pockets with a two-step similarity-based docking protocol. This procedure exploits a significant structural conservation of ligand binding modes across remote homologs [17]. First, globally similar ligand-bound templates from the PDB, identified by *e*FindSite to have a similar pocket as the BindingDB protein, are superimposed onto the apo-model. Proteins are aligned with Fr-TM-align [18] employing the Template Modeling score (TM-score) [19] to measure the global structure similarity. Subsequently, the BindingDB compound is aligned onto the template-bound ligand in order to place it in the predicted pocket of the apo-model. Here, we use chemical alignments constructed with kcombu [20], which also reports the chemical similarity between the BindingDB compound and the template-bound ligand measured by the Tanimoto coefficient (TC). Since a perfect case corresponds to both a TM-score and a TC of 1.0, we

introduce a new metric, the Perfect Match Distance (PMD), combining protein structure and ligand chemical similarity values:

$$PMD = \sqrt{(1 - TM\text{-}score)^2 + (1 - TC)^2} \quad \text{Eq. 1.}$$

PMD is simply the Cartesian distance from the perfect match in the TM-score/TC space. In order to generate only high-quality holo-models, those cases with a PMD of >0.6 are excluded from the modeling process. This PMD cutoff was chosen to ensure that TM-score and TC for the selected templates are always above their individual significance threshold values of 0.4 [19, 20]. Further, for those cases having multiple ligand-bound templates satisfying the PMD criterion of ≤0.6, a template with the shortest PMD is selected to build the holo-model of the BindingDB complex.

Complex structure refinement and assessment

In the final phase, protein models are rebuilt in the presence of the docked BindingDB compounds with Modeller. To make sure that the binding site is remodeled to accommodate the specific ligand, binding residues identified by *e*FindSite are removed from the initial model while enforcing the presence of secondary structure predicted by PSIPRED [21]. The resulting models are further annotated with the ligand-protein interaction score according to the Distance-scaled Finite Ideal-gas REference (DFIRE) potential [22]. The *e*Model-BDB database contains atomic-level structure models of 200,005 drug-protein interactions from BindingDB, comprising 2,791 non-redundant proteins and 108,363 drug-like compounds. The list of *e*Model-BDB complexes is provided as Supplementary File S1.

Analyses

Data quality control

The quality control is pertinent to both protein structure modeling as well as binding site prediction. The quality of protein models is assessed with ModelEvaluator employing various structural features to compute the absolute quantitative score with a support vector

regression. This approach assigns the GDT-score to a model by analyzing its secondary structure, relative solvent accessibility, contact map, and β-sheet structure. It has been demonstrated that GDT-scores estimated by ModelEvaluator for template-based models are highly correlated with the actual values with the Pearson correlation coefficient of 0.82 [12]. The first violin in Figure 2 shows that *e*Model-BDB contains close and remote homology models with the median target-template sequence identity of 63%. The second violin indicates that the vast majority of these structures are accurate with the median estimated GDT-score for BindingDB proteins is 0.62. Further, as many as 78% of binding sites predicted by *e*FindSite to match BindingDB ligands have a high confidence of >0.8. We showed previously that confidence scores of >0.8 assigned by *e*FindSite correspond to the Matthews correlation coefficient (MCC) [23] of ≥0.6 for predicted binding residues [15]. On that account, we expect that the majority of binding sites for BindingDB drugs are correctly annotated as well. Note that in contrast to other pocket predictors, *e*FindSite annotations and confidence assignments are, to some extent, independent of the accuracy of protein models.

The quality of complex models is controlled by imposing thresholds on the chemical similarity between BindingDB and PDB ligands as well as the global structure similarity between *e*Thread models and ligand-bound templates from the PDB. Figure 3A shows the distribution of both parameters across *e*Model-BDB models. Encouragingly, the median TM-score and TC for ligand-bound templates used to build *e*Model-BDB are as high as 0.81 and 0.67, respectively. Previous studies show that the probability for a protein pair to belong to the same fold is 98% when the TM-score is close to 0.8 [24]. In addition, it was demonstrated that the root-mean-square deviation (RMSD) over ligand non-hydrogen atoms for similarity-based docking conducted with the TC in the range of 0.6-0.8 is typically 2-3 Å [25]. TM-score and TC values are combined into a single assessment score, the PMD, measuring the distance from the perfect match. Therefore, selecting template proteins with a lower TM-score to BindingDB targets requires their ligands to have a high TC and vice versa, selecting PDB ligands with a lower chemical similarity to BindingDB molecules requires a high global structure similarity between proteins. Figure 3B shows that the median PMD for *e*Model-BDB complex models is 0.46.

Data validation

In addition to the rigorous quality control maintained during dataset generation, *e*Model-BDB is validated retrospectively against experimental structures recently deposited to the PDB. The structure models of BindingDB interactions have been constructed with the PDB library as of January 31st 2017, therefore, to validate *e*Model-BDB models, 7,012 experimental structures deposited to the PDB after February 2017 were considered. The validation protocol is made more challenging by including only remote homology models with a template-target sequence identity of <40%. In order to maximize the validation coverage, we use the recently determined structures of *e*Model-BDB targets and their homologs with at least 40% sequence identity. After applying these filters, 41 recently solved experimental structures selected from the PDB can be used to validate 161 *e*Thread models and 952 BindingDB reaction set IDs, comprising 39 target proteins, 52 pockets, and 881 compounds. This set is referred to as the validation dataset. The list of validation pairs is given in Supplementary File S2.

Protein structure modeling

The first violin in Figure 4 shows that the median TM-score of *e*Model-BDB vs. experimental structures is 0.85 with as many as 98.1% of the models having a TM-score of ≥0.4. Clearly, the majority of structures are modeled by *e*Thread with a high accuracy. A representative example of the correctly predicted target structure is dihydrofolate reductase (DHFR) from *Streptococcus pyogenes* build on the crystal structure of DHFR from *Streptococcus pneumoniae* (PDB-ID: 3ix9, chain B, 36% sequence identity to the target) [26]. The *e*Thread model, whose estimated GDT-score is 0.92, was then used to construct a structure model for the BindingDB reactant set ID 00267770 consisting of DHFR complexed with BDBM50329610. This model is validated against the crystal structure of DHFR-UCP1106 from *Staphylococcus aureus* (PDB-ID: 5isp, chain X, 43% sequence identity to the target) released on 2017-06-28 [27]. Figure 5 shows the predicted weakly homologous model of DHFR-BDBM50329610 (purple) superposed on the experimental structure of DHFR-UCP1106 (gold). The *e*Model-BDB model is indeed highly accurate with a TM-score of 0.95 and a Cα-RMSD of 1.23 Å over 157 aligned residues. In addition, Figure 6A shows that the estimated GDT-score employed in this study as

the confidence measure to control the quality of protein models correlates with the accuracy of final models evaluated with the TM-score. On that account, the estimated GDT-score provides a robust quality assessment measure to control the quality of models in *e*Model-BDB.

Binding pocket prediction

The accuracy of pocket prediction is validated by superposing the experimental holo structure onto the *e*Model-BDB model and then calculating the distance between the geometric center of a bound ligand in the experimental complex and the pocket center predicted by *e*FindSite in the model. The second violin in Figure 4 shows that the median pocket distance is 5.5 Å with 59.6% of pockets predicted within 6 Å, therefore, most *e*FindSite annotations are accurate. A binding site in the model of vitamin D receptor (VDR) is a representative example of a pocket predicted with *e*FindSite. This model was constructed for the BindingDB reactant set ID 50662356 based on human retinoic acid receptor RXR-alpha (PDB-ID: 4nqa, chain H, 38% sequence identity to the target) [28]. Although the GDT-score estimated for the VDR model is 0.62 indicating a moderately accurate structure, the top-ranked binding site annotated by *e*FindSite is assigned a high confidence of 94.2%. Figure 7 shows the VDR model (purple ribbons) superposed onto the crystal structure of vitamin D3 receptor A (gold ribbons) complexed with a synthetic analog of 1α,25-dihydroxyvitamin D3 (PDB-ID: 5nky, chain A, 66% sequence identity to the target) released on 2017-05-24 [29]. Not only the VDR model aligns well to the experimental structure with a TM-score of 0.90 and a Cα-RMSD of 2.13 Å over 235 residues, but also the predicted pocket center (purple sphere) is only 5.5 Å away from the geometric center of vitamin D analog (gold sphere).

Ligand docking

Finally, we calculate the RMSD over non-hydrogen atoms between the BindingDB drug in the *e*Model-BDB structure and the bound ligand in the superposed experimental complex. Here, we employ a subset of 37 models selected from the validation set whose pocket centers are predicted within 8 Å. The first violin in Figure 8 shows that the median ligand RMSD is 2.6 Å and it is $\leq$3 Å for 58.1% of BindingDB compounds. The model of the BindingDB reactant set ID

50974033 consisting of tyrosine-protein kinase (TSK) complexed with BDBM50399512 is selected to exemplify the accuracy of complex structures in *e*Model-BDB. The model of TSK built on the crystal structure of human hemopoietic cell kinase (HCK) (PDB-ID: 1qcf, chain A, 39% sequence identity to the target) [30] by *e*Thread is assigned an estimated GDT-score of 0.61. Subsequently, the complex model of TSK-BDBM50399512 was constructed by similarity-based docking employing the crystal structure of HCK bound to a pyrazolo-pyrimidine inhibitor (PDB-ID: 3vs7, chain B, 37% sequence identity to the target) [31]. This HCK complex was selected as the best ligand-bound template based on the high TM-score of 0.64 and TC of 0.53, yielding the shortest PMD of 0.35. Figure 9 shows the validation of the modeled TSK-BDBM50399512 by the experimental structure of Bruton's tyrosine kinase (Btk) bound to an anti-cancer drug, ibrutinib (PDB-ID: 5p9i, chain A, TM-score: 0.92, 58% sequence identity to the target) released on 2017-05-24 [32]. Kcombu reports a significant chemical alignment between BDBM50399512 and ibrutinib with a TC of 0.68 (Figure 9A). Upon the superposition of TSK and Btk proteins, the RMSD between BDBM50399512 docked to the model and ibrutinib bound in the experimental structure calculated over the chemical alignment reported by kcombu is 2.62 Å (Figure 9B). These results verify that the computer-generated TSK-BDBM50399512 model for the BindingDB reactant set ID 50974033 is correct.

The model of the BindingDB reactant set ID 50103430 consisting of cytochrome P450 17A1 (CYP17A1) complexed with BDBM50061174 is selected to exemplify the accuracy of complex structures in *e*Model-BDB. The model of CYP17A1 built on the crystal structure of human microsomal cytochrome P450 2A6 (PDB-ID: 1z11, chain A, 29% sequence identity to the target) [33] by *e*Thread is assigned an estimated GDT-score of 0.69. Subsequently, the complex model of CYP17A1-BDBM50061174 was constructed by similarity-based docking employing the crystal structure of CYP17A1 bound to abiraterone, a steroidal prostate cancer drug (PDB-ID: 3ruk, chain D) [34]. The CYP17A1-abiraterone complex was selected as the best ligand-bound template based on the high TM-score of 0.84 and TC of 0.89, yielding the shortest PMD of 0.19. Figure 9 shows the validation of the modeled CYP17A1-BDBM50061174 by the experimental structure of CYP17A1-(R)-orteronel (PDB-ID: 5irq, chain B, 64% sequence identity to the target) released on 2017-03-15 [35]. Kcombu reports a significant chemical alignment between

steroidal BDBM50061174 and nonsteroidal (R)-orteronel with a TC of 0.54 (Figure 9A). Upon the superposition of CYP17A1 proteins, the RMSD between BDBM50061174 docked to the model and (R)-orteronel bound in the experimental structure calculated over the chemical alignment reported by kcombu is 2.95 Å (Figure 9B). These results verify that the computer-generated CYP17A1-BDBM50061174 model for the BindingDB reactant set ID 50103430 is correct.

Similarity-based docking procedure employed to construct ligand-bound structures in *e*Model-BDB superposes target ligands onto template molecules selected from the PDB according to the chemical alignment reported by kcombu. One may expect that superposing target compounds onto chemically similar template ligands yields more accurate binding poses than those generated from chemically less similar template molecules. Indeed, Figure 6B shows that the target-template chemical similarity measured with the TC correlates with the docking accuracy evaluated with the RMSD of ligand poses constructed based on target-template alignments. These results are in line with other studies reporting that the average RMSD values for similarity-based docking methods are generally below 2 Å when the target-template similarities are above 0.7 [36]. The performance of similarity-based docking employed to construct *e*Model-BDB is also compared to that of AutoDock Vina [37] and rDock [38]. In contrast to the median ligand RMSD of 2.6 Å for *e*Model-BDB complexes, the median RMSD values for BindingDB drugs docked to *e*FindSite pockets with AutoDock Vina and rDock are 6.7 Å and 7.2 Å, respectively (Figure 8). We note that similarity-based docking was demonstrated to outperform traditional docking when the target-template similarity is greater than 0.4 [36], which was employed as the TC threshold to construct *e*Model-BDB complex models. Overall, the quality assessment as well as independently obtained validation results demonstrate that the *e*Model-BDB database contains high-quality models closely resembling experimentally determined structures, not only at the global structure level, but also at the level of binding pockets and bound ligands.

Discussion

*e*Model-BDB is generated to support rational drug development projects. These data can directly aid structure-based drug discovery pipelines and protein function analysis by providing atomic-level models of a large set of drug-protein interactions with known affinities curated in the BindingDB. An important application of *e*Model-BDB is computational drug repositioning, i.e. finding new indications for existing drugs [39]. Although drug repurposing holds a significant promise to speed up drug development, particularly for diseases considered to be unprofitable, its major bottleneck is the scarce structural information on druggable pockets. On that account, a diverse dataset of small, drug-like molecules bound to high-quality models with accurately annotated pockets provide an invaluable resource for drug repositioning employing sequence order-independent pocket matching algorithms [40-43]. It is noteworthy that computational drug repurposing has suggested new opportunities to combat tuberculosis [44, 45], malaria [46], and rare diseases [47, 48].

Binding sites in *e*Model-BDB can also be matched to pockets predicted in potential drug targets in order to determine whether these proteins are druggable or not. If a new pocket aligns well with drug-bound pockets in *e*Model-BDB then it is likely going to be druggable. That being the case, our data can be utilized right at the outset of drug discovery, in the target identification phase. Finally, ligand binding can significantly help stabilize a protein, particularly from the point of view of the conformational stability [49]. *e*Model-BDB can, therefore, inform crystallography efforts by suggesting possible compounds binding to certain protein targets at either the active or allosteric sites in order to increase the chances of successful crystallization.

Availability of supporting data and materials

Structure models in *e*Model-BDB are named according to the BindingDB reactant set IDs, which can be obtained by searching the BindingDB at https://www.bindingdb.org. This procedure is illustrated in Figure 10. The BindingDB can be searched either by protein and compound names (Figure 10A) or by the target sequence (Figures 10B and 10C). Next, the complex of interest can be selected from the list of hits (Figure 10D) in order to download the corresponding SDfile of the complex (Figure 10E). The BindingDB reactant set ID, e.g. 00267770, is stored inside the SDfile (Figure 10F). The reactant set ID can then be used to find the detailed information on the

BindingDB website, e.g. https://www.bindingdb.org/jsp/dbsearch/Summary_ki.jsp?reactant_set_id=00267770 (Figure 10G) as well as access the structure model in *e*Model-BDB, e.g. http://brylinski.cct.lsu.edu/pub/eModelBDB.php?reactant_set_id=00267770 (Figure 10H). Data is also available to download from the *GigaScience* GigaDB database [50].

Declarations

List of abbreviations

BindingDB: Binding Database; DFIRE: Distance-scaled Finite Ideal-gas REference; GDT-score: Global Distance Test score; MCC: Matthews correlation coefficient; PMD: Perfect Match Distance; PDB: Protein Data Bank; RMSD: root-mean-square deviation; TC: Tanimoto coefficient; TM-score: Template Modeling score

Competing interests

The authors declare that they have no competing interests.

Funding

Research reported in this publication was supported by the National Institute of General Medical Sciences of the National Institutes of Health under Award Number R35GM119524.

Authors' contributions

MB prepared protein models, annotated binding pockets, and validated protein structures and pockets. MN constructed, refined, and validated drug-bound models. RGG prepared case studies. MN and MB wrote the paper.

Acknowledgements

Authors are grateful to Louisiana State University for providing computing resources.

References

1. O'Leary NA, Wright MW, Brister JR, Ciufo S, Haddad D, McVeigh R, et al. Reference sequence (RefSeq) database at NCBI: current status, taxonomic expansion, and functional annotation. Nucleic Acids Res. 2016;44 D1:D733-45. doi:10.1093/nar/gkv1189.
2. Berman HM, Westbrook J, Feng Z, Gilliland G, Bhat TN, Weissig H, et al. The Protein Data Bank. Nucleic Acids Res. 2000;28 1:235-42.
3. Liu T, Lin Y, Wen X, Jorissen RN and Gilson MK. BindingDB: a web-accessible database of experimentally determined protein-ligand binding affinities. Nucleic Acids Res. 2007;35 Database issue:D198-201. doi:10.1093/nar/gkl999.
4. Castrignano T, De Meo PD, Cozzetto D, Talamo IG and Tramontano A. The PMDB Protein Model Database. Nucleic Acids Res. 2006;34 Database issue:D306-9. doi:10.1093/nar/gkj105.
5. Sanchez R, Pieper U, Mirkovic N, de Bakker PI, Wittenstein E and Sali A. MODBASE, a database of annotated comparative protein structure models. Nucleic Acids Res. 2000;28 1:250-3.
6. Brylinski M and Lingam D. eThread: a highly optimized machine learning-based approach to meta-threading and the modeling of protein tertiary structures. PLoS One. 2012;7 11:e50200. doi:10.1371/journal.pone.0050200.
7. Sali A and Blundell TL. Comparative protein modelling by satisfaction of spatial restraints. J Mol Biol. 1993;234 3:779-815. doi:10.1006/jmbi.1993.1626.
8. Remmert M, Biegert A, Hauser A and Soding J. HHblits: lightning-fast iterative protein sequence searching by HMM-HMM alignment. Nat Methods. 2011;9 2:173-5. doi:10.1038/nmeth.1818.
9. Yang Y, Faraggi E, Zhao H and Zhou Y. Improving protein fold recognition and template-based modeling by employing probabilistic-based matching between predicted one-dimensional structural properties of query and corresponding native properties of templates. Bioinformatics. 2011;27 15:2076-82. doi:10.1093/bioinformatics/btr350.
10. Ma J, Wang S, Zhao F and Xu J. Protein threading using context-specific alignment potential. Bioinformatics. 2013;29 13:i257-65. doi:10.1093/bioinformatics/btt210.
11. Xu D and Zhang Y. Improving the physical realism and structural accuracy of protein models by a two-step atomic-level energy minimization. Biophys J. 2011;101 10:2525-34. doi:10.1016/j.bpj.2011.10.024.
12. Wang Z, Tegge AN and Cheng J. Evaluating the absolute quality of a single protein model using structural features and support vector machines. Proteins. 2009;75 3:638-47. doi:10.1002/prot.22275.
13. He Z, Alazmi M, Zhang J and Xu D. Protein structural model selection by combining consensus and single scoring methods. PLoS One. 2013;8 9:e74006. doi:10.1371/journal.pone.0074006.
14. Mirabello C, Adelfio A and Pollastri G. Reconstructing protein structures by neural network pairwise interaction fields and iterative decoy set construction. Biomolecules. 2014;4 1:160-80. doi:10.3390/biom4010160.

15. Brylinski M and Feinstein WP. eFindSite: improved prediction of ligand binding sites in protein models using meta-threading, machine learning and auxiliary ligands. J Comput Aided Mol Des. 2013;27 6:551-67. doi:10.1007/s10822-013-9663-5.
16. Feinstein WP and Brylinski M. eFindSite: Enhanced fingerprint-based virtual screening against predicted ligand binding sites in protein models. Mol Inform. 2014;33 2:135-50. doi:10.1002/minf.201300143.
17. Brylinski M and Skolnick J. FINDSITE: a threading-based approach to ligand homology modeling. PLoS Comput Biol. 2009;5 6:e1000405. doi:10.1371/journal.pcbi.1000405.
18. Pandit SB and Skolnick J. Fr-TM-align: a new protein structural alignment method based on fragment alignments and the TM-score. BMC Bioinformatics. 2008;9:531. doi:10.1186/1471-2105-9-531.
19. Zhang Y and Skolnick J. Scoring function for automated assessment of protein structure template quality. Proteins. 2004;57 4:702-10. doi:10.1002/prot.20264.
20. Kawabata T. Build-up algorithm for atomic correspondence between chemical structures. Journal of chemical information and modeling. 2011;51 8:1775-87. doi:10.1021/ci2001023.
21. Jones DT. Protein secondary structure prediction based on position-specific scoring matrices. J Mol Biol. 1999;292 2:195-202. doi:10.1006/jmbi.1999.3091.
22. Zhang C, Liu S, Zhu Q and Zhou Y. A knowledge-based energy function for protein-ligand, protein-protein, and protein-DNA complexes. J Med Chem. 2005;48 7:2325-35. doi:10.1021/jm049314d.
23. Matthews BW. Comparison of the predicted and observed secondary structure of T4 phage lysozyme. Biochim Biophys Acta. 1975;405 2:442-51.
24. Xu J and Zhang Y. How significant is a protein structure similarity with TM-score = 0.5? Bioinformatics. 2010;26 7:889-95. doi:10.1093/bioinformatics/btq066.
25. Brylinski M. Nonlinear scoring functions for similarity-based ligand docking and binding affinity prediction. Journal of chemical information and modeling. 2013;53 11:3097-112. doi:10.1021/ci400510e.
26. Lee J, Yennawar NH, Gam J and Benkovic SJ. Kinetic and structural characterization of dihydrofolate reductase from Streptococcus pneumoniae. Biochemistry. 2010;49 1:195-206. doi:10.1021/bi901614m.
27. Reeve SM, Scocchera E, Ferreira JJ, N GD, Keshipeddy S, Wright DL, et al. Charged propargyl-linked antifolates reveal mechanisms of antifolate resistance and inhibit trimethoprim-resistant MRSA strains possessing clinically relevant mutations. J Med Chem. 2016;59 13:6493-500. doi:10.1021/acs.jmedchem.6b00688.
28. Lou X, Toresson G, Benod C, Suh JH, Philips KJ, Webb P, et al. Structure of the retinoid X receptor alpha-liver X receptor beta (RXRalpha-LXRbeta) heterodimer on DNA. Nat Struct Mol Biol. 2014;21 3:277-81. doi:10.1038/nsmb.2778.
29. Belorusova AY, Martinez A, Gandara Z, Gomez G, Fall Y and Rochel N. Structure-activity relationship study of vitamin D analogs with oxolane group in their side chain. Eur J Med Chem. 2017;134:86-96. doi:10.1016/j.ejmech.2017.03.081.
30. Schindler T, Sicheri F, Pico A, Gazit A, Levitzki A and Kuriyan J. Crystal structure of Hck in complex with a Src family-selective tyrosine kinase inhibitor. Mol Cell. 1999;3 5:639-48.

31. Saito Y, Yuki H, Kuratani M, Hashizume Y, Takagi S, Honma T, et al. A pyrrolo-pyrimidine derivative targets human primary AML stem cells in vivo. Sci Transl Med. 2013;5 181:181ra52. doi:10.1126/scitranslmed.3004387.
32. Bender AT, Gardberg A, Pereira A, Johnson T, Wu Y, Grenningloh R, et al. Ability of Bruton's Tyrosine Kinase Inhibitors to Sequester Y551 and Prevent Phosphorylation Determines Potency for Inhibition of Fc Receptor but not B-Cell Receptor Signaling. Mol Pharmacol. 2017;91 3:208-19. doi:10.1124/mol.116.107037.
33. Yano JK, Hsu MH, Griffin KJ, Stout CD and Johnson EF. Structures of human microsomal cytochrome P450 2A6 complexed with coumarin and methoxsalen. Nat Struct Mol Biol. 2005;12 9:822-3. doi:10.1038/nsmb971.
34. DeVore NM and Scott EE. Structures of cytochrome P450 17A1 with prostate cancer drugs abiraterone and TOK-001. Nature. 2012;482 7383:116-9. doi:10.1038/nature10743.
35. Petrunak EM, Rogers SA, Aube J and Scott EE. Structural and functional evaluation of clinically relevant inhibitors of steroidogenic cytochrome P450 17A1. Drug Metab Dispos. 2017;45 6:635-45. doi:10.1124/dmd.117.075317.
36. Kawabata T and Nakamura H. 3D flexible alignment using 2D maximum common substructure: dependence of prediction accuracy on target-reference chemical similarity. Journal of chemical information and modeling. 2014;54 7:1850-63. doi:10.1021/ci500006d.
37. Trott O and Olson AJ. AutoDock Vina: improving the speed and accuracy of docking with a new scoring function, efficient optimization, and multithreading. J Comput Chem. 2010;31 2:455-61. doi:10.1002/jcc.21334.
38. Ruiz-Carmona S, Alvarez-Garcia D, Foloppe N, Garmendia-Doval AB, Juhos S, Schmidtke P, et al. rDock: a fast, versatile and open source program for docking ligands to proteins and nucleic acids. PLoS Comput Biol. 2014;10 4:e1003571. doi:10.1371/journal.pcbi.1003571.
39. Haupt VJ and Schroeder M. Old friends in new guise: repositioning of known drugs with structural bioinformatics. Brief Bioinform. 2011;12 4:312-26. doi:10.1093/bib/bbr011.
40. Brylinski M. eMatchSite: sequence order-independent structure alignments of ligand binding pockets in protein models. PLoS Comput Biol. 2014;10 9:e1003829. doi:10.1371/journal.pcbi.1003829.
41. Brylinski M. Local alignment of ligand binding sites in proteins for polypharmacology and drug repositioning. Methods Mol Biol. 2017;1611:109-22. doi:10.1007/978-1-4939-7015-5_9.
42. Konc J and Janezic D. ProBiS algorithm for detection of structurally similar protein binding sites by local structural alignment. Bioinformatics. 2010;26 9:1160-8. doi:10.1093/bioinformatics/btq100.
43. Ren J, Xie L, Li WW and Bourne PE. SMAP-WS: a parallel web service for structural proteome-wide ligand-binding site comparison. Nucleic Acids Res. 2010;38 Web Server issue:W441-4. doi:10.1093/nar/gkq400.
44. Kinnings SL, Liu N, Buchmeier N, Tonge PJ, Xie L and Bourne PE. Drug discovery using chemical systems biology: repositioning the safe medicine Comtan to treat multi-drug

and extensively drug resistant tuberculosis. PLoS Comput Biol. 2009;5 7:e1000423. doi:10.1371/journal.pcbi.1000423.

45. Anand P and Chandra N. Characterizing the pocketome of Mycobacterium tuberculosis and application in rationalizing polypharmacological target selection. Sci Rep. 2014;4:6356. doi:10.1038/srep06356.

46. Ramakrishnan G, Chandra N and Srinivasan N. Exploring anti-malarial potential of FDA approved drugs: an in silico approach. Malar J. 2017;16 1:290. doi:10.1186/s12936-017-1937-2.

47. Brylinski M, Naderi M, Govindaraj RG and Lemoine J. eRepo-ORP: Exploring the opportunity space to combat orphan diseases with existing drugs. J Mol Biol. 2018;in press doi:10.1016/j.jmb.2017.12.001.

48. Govindaraj RG, Naderi M, Singha M, Lemoine J and Brylinski M. Large-scale computational drug repositioning to find treatments for rare diseases. NPJ Syst Biol Appl. 2018;4:13. doi:10.1038/s41540-018-0050-7.

49. Deller MC, Kong L and Rupp B. Protein stability: a crystallographer's perspective. Acta Crystallogr F Struct Biol Commun. 2016;72 Pt 2:72-95. doi:10.1107/S2053230X15024619.

50. Naderi M; Govindaraj RG; Brylinski M (2018): Supporting data for "eModel-BDB: A database of comparative structure models of drug-target interactions from the Binding Database" GigaScience Database. http://dx.doi.org/100396

Figure captions

Figure 1. Deposition rate of ligand-bound structures to the Protein Data Bank. The total number of protein chains binding small molecules (light gray squares and a dashed line) is counted at any point in time. The number of unique complex structures is obtained by clustering individual chains at 80% sequence identity (dark gray circles and a solid line). N_t and N_u in the linear regression equations are the total and unique number of ligand-protein complexes, respectively, and *m* stands for month.

Figure 2. Violin and box plots for model quality control. The distribution of the target-template sequence identity (SeqId) and the Global Distance Test (GDT) score estimated for structure models. Horizontal yellow lines represent median values.

Figure 3. Similarities between target and holo-template proteins. (**A**) The chemical similarity between BindingDB and PDB ligands measured with the Tanimoto coefficient (TC) is plotted against the global structure similarity of *e*Thread models and ligand-bound templates from the

PDB assessed by the TM-score. The 2D contour plot is generated by smoothing the data with the kernel density estimation technique. 1D histograms show the distribution of TC (top) and TM-score (right) values across *e*Model-BDB models. (**B**) Violin and box plot for the holo-template Perfect Match Distance (PMD) combining TC and TM-score. The horizontal yellow line represents the median value.

Figure 4. Violin and box plots for the distribution of validation scores. The accuracy is assessed for remote homology complex models in the validation set. The global structure similarity is measured with the TM-score. The pocket distance is measured between the predicted pocket center and the geometric center of the ligand in the experimental structure superposed onto the *e*Thread model. Horizontal yellow lines represent median values.

Figure 5. Representative example of a structure model constructed by *e*Thread. The model of dihydrofolate reductase (DHFR, purple ribbons) complexed with BDBM50329610 is superposed onto the crystal structure of homologous DHFR from *S. aureus* (gold ribbons) complexed with UCP1106. Ligands bound to target proteins are shown as solid sticks (BDBM50329610 is purple and UCP1106 is gold) with non-carbon atoms colored by atom type (O – red, N – blue).

Figure 6. Analysis of structure modeling and ligand docking accuracy. The accuracy is assessed for remote homology complex models in the validation set. (**A**) Accuracy of global structure prediction evaluated by the TM-score with respect to the estimated GDT-score. (**B**) Accuracy of similarity-based docking with respect to the chemical similarity between BindingDB and PDB ligands measured by the Tanimoto coefficient (TC). The ligand RMSD is calculated over non-hydrogen atoms according to the chemical alignment reported by kcombu. Solid red lines show the average prediction accuracy for binned GDT-score values in **A** and the chemical similarity in **B**. Dotted black lines mark the median TM-score in **A** and RMSD in **B** across all benchmarking cases.

Figure 7. Representative example of a binding site detected by *e*FindSite. The model of vitamin D receptor (VDR, purple ribbons) is superposed onto the crystal structure of homologous VDR from human (gold ribbons) complexed with a synthetic analog of vitamin D (gold and red sticks). Cα atoms of binding residues predicted in the VDR model by *e*FindSite are shown as small spheres. Large spheres connected by a dashed black line are placed at the location of the predicted pocket center (purple) and the geometric center of vitamin D analog (gold).

Figure 8. Violin and box plots for the docking accuracy. The accuracy is assessed for remote homology complex models in the validation set. The ligand RMSD is calculated over non-hydrogen atoms according to the chemical alignment reported by kcombu. The performance of similarity-based docking employed to construct *e*Model-BDB is compared to that of AutoDock Vina and rDock. Horizontal yellow lines represent median values.

Figure 9. Representative example of a complex structure constructed by similarity-based docking. (**A**) Chemical alignment between BDBM50399512 (left) and ibrutinib (right) reported by kcombu. 26 equivalent atom pairs constituting the maximum common substructure are outlined in purple in BDBM50399512 and in gold in ibrutinib. (**B**) The model of tyrosine-protein kinase (TSK, purple ribbons) is superposed onto the crystal structure of HCK (gold ribbons) complexed with ibrutinib (gold sticks). Cα atoms of binding residues identified in the TSK model by *e*FindSite are shown as purple spheres, whereas the target compound, BDBM50399512, docked into the predicted pocket is represented by purple sticks. Non-carbon atoms in BDBM50399512 and ibrutinib are colored by atom type (O – red, N – blue, F – green).

Figure 10. Procedure to obtain *e*Model-BDB complexes via the BindingDB website. Target complex can be identified based on either the protein (red arrows and boxes) or the ligand of interest (blue arrows and boxes). Common actions that a user needs to perform are colored in green. (**A**) Specific ligands and proteins can directly be searched for on the BindingDB website. (**B**, **C**) Alternatively, target proteins can be found with the blast search. (**D**) A complex of interest

can then be selected in order to (**E**) generate and download a SDfile. (**F**) The BindingDB reactant set ID stored inside the SDfile is used to (**G**) view a web page containing detailed information about the target complex as well as (**H**) access the corresponding *e*Model-BDB structure model named according to the BindingDB reactant set ID.

Figure 1

Click here to access/download;Figure;Figure1.tif

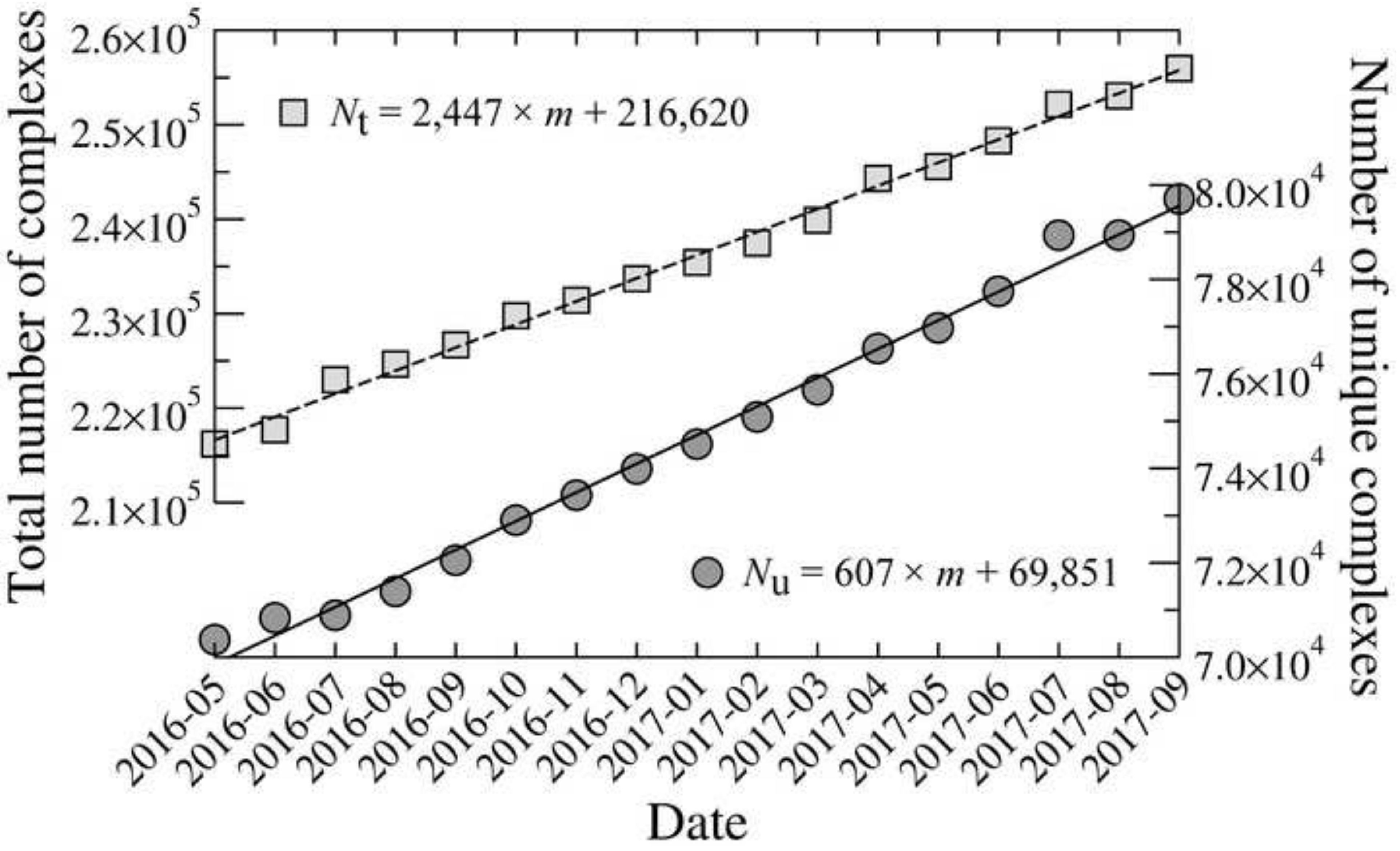

Figure 2

Click here to access/download;Figure;Figure2.tif

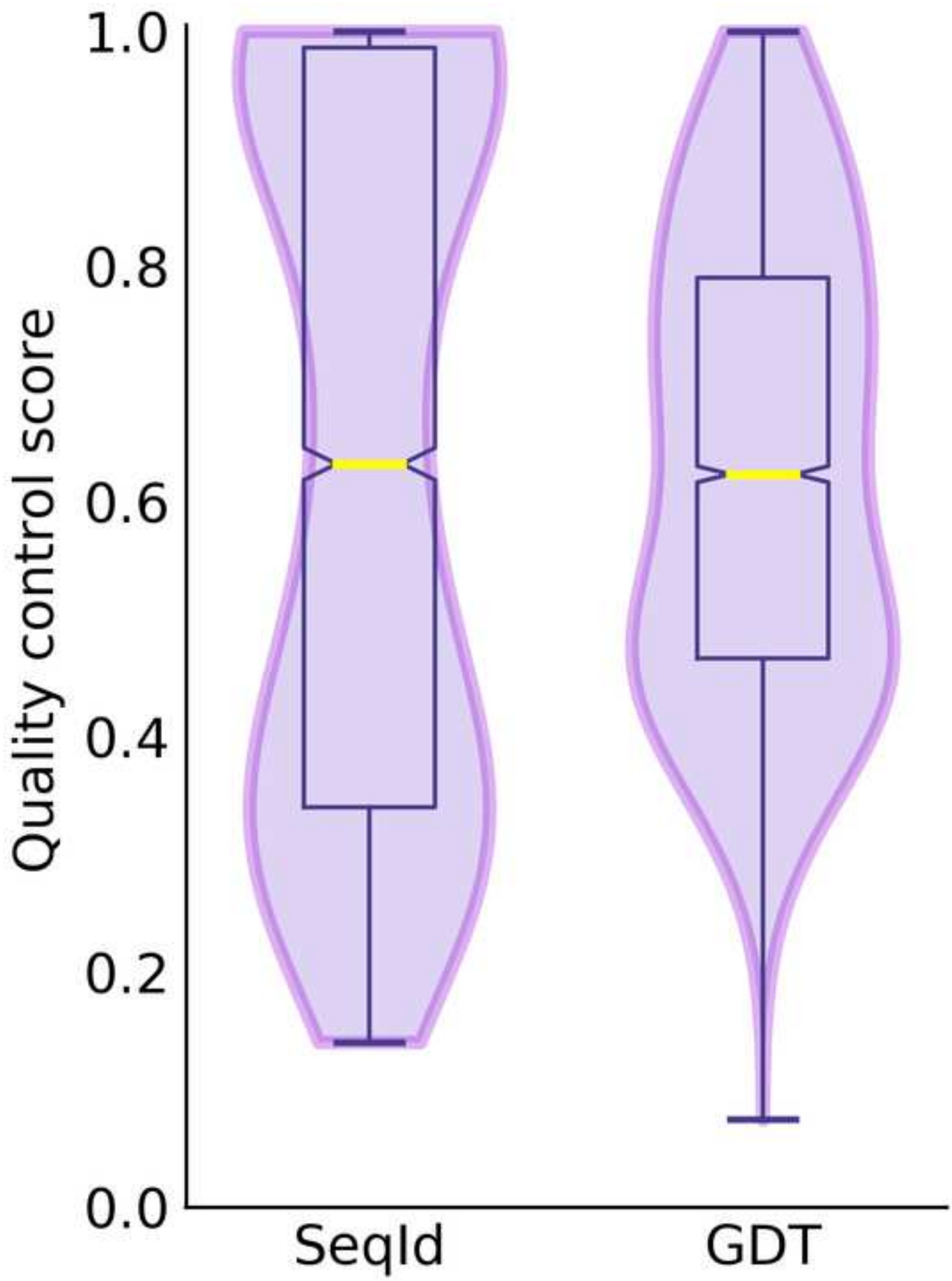

Figure 3

Click here to access/download;Figure;Figure3.tif

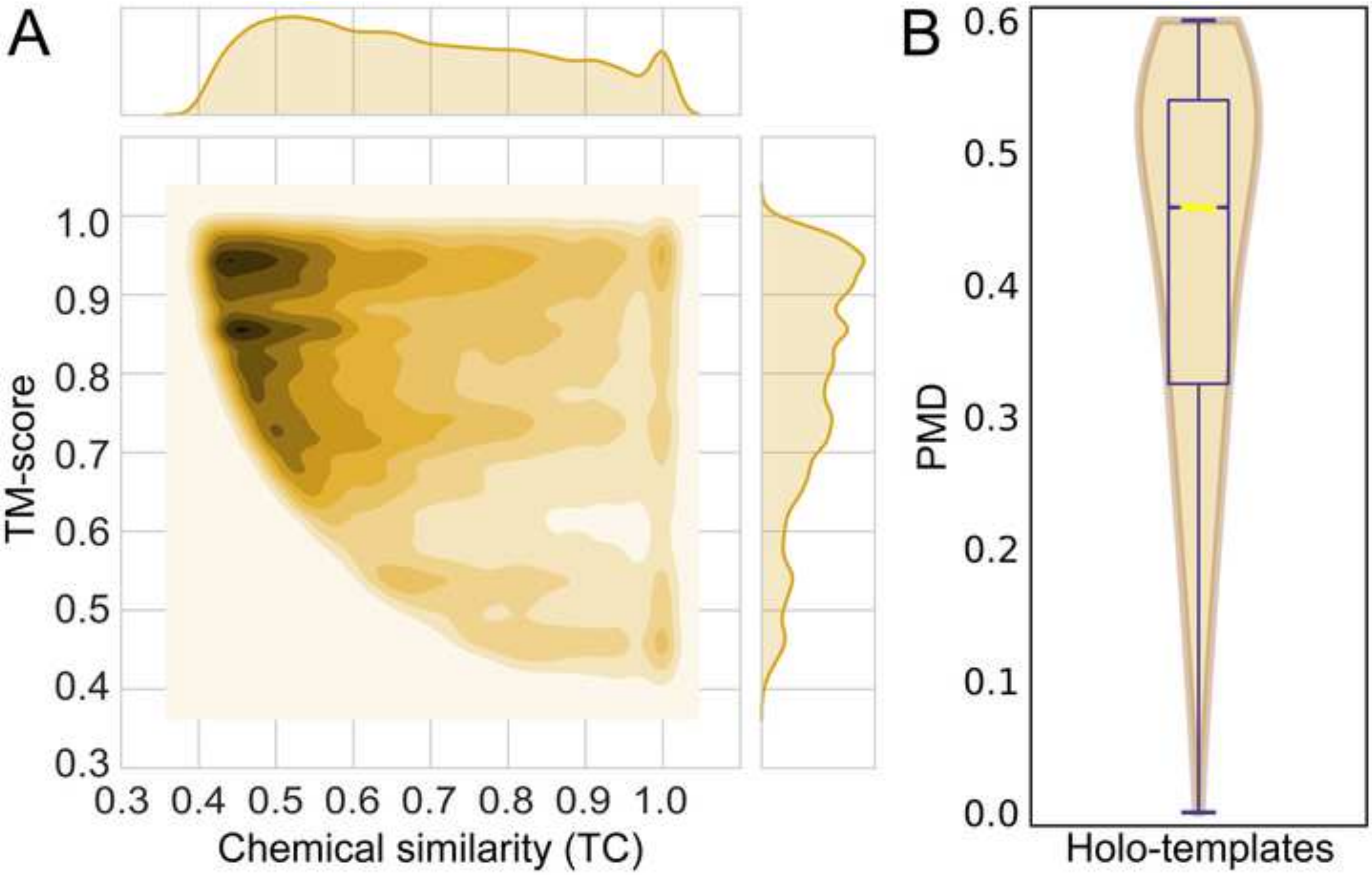

Figure 4 Click here to access/download;Figure;Figure4.tif

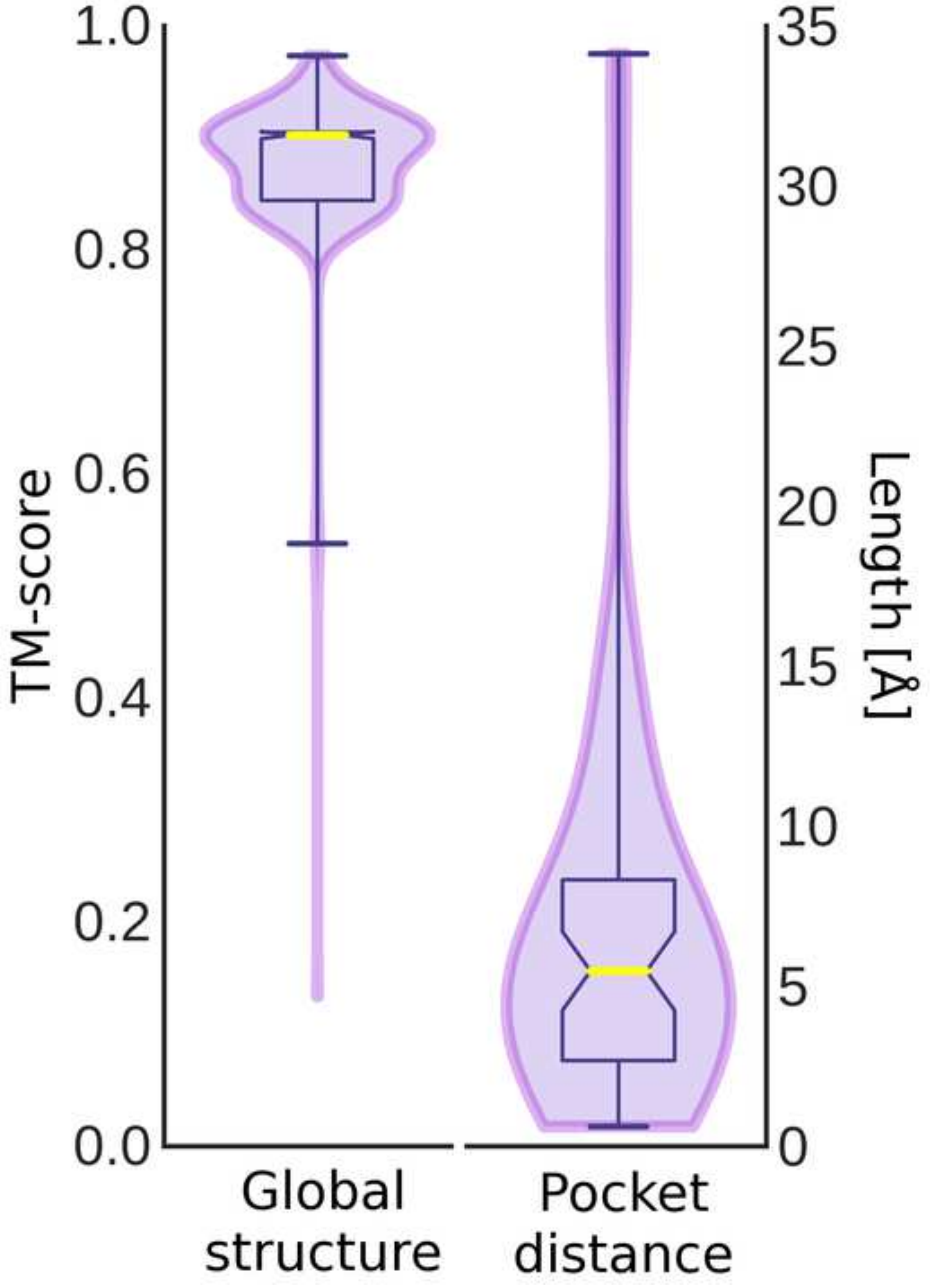

Figure 5

Click here to access/download;Figure;Figure5.tif

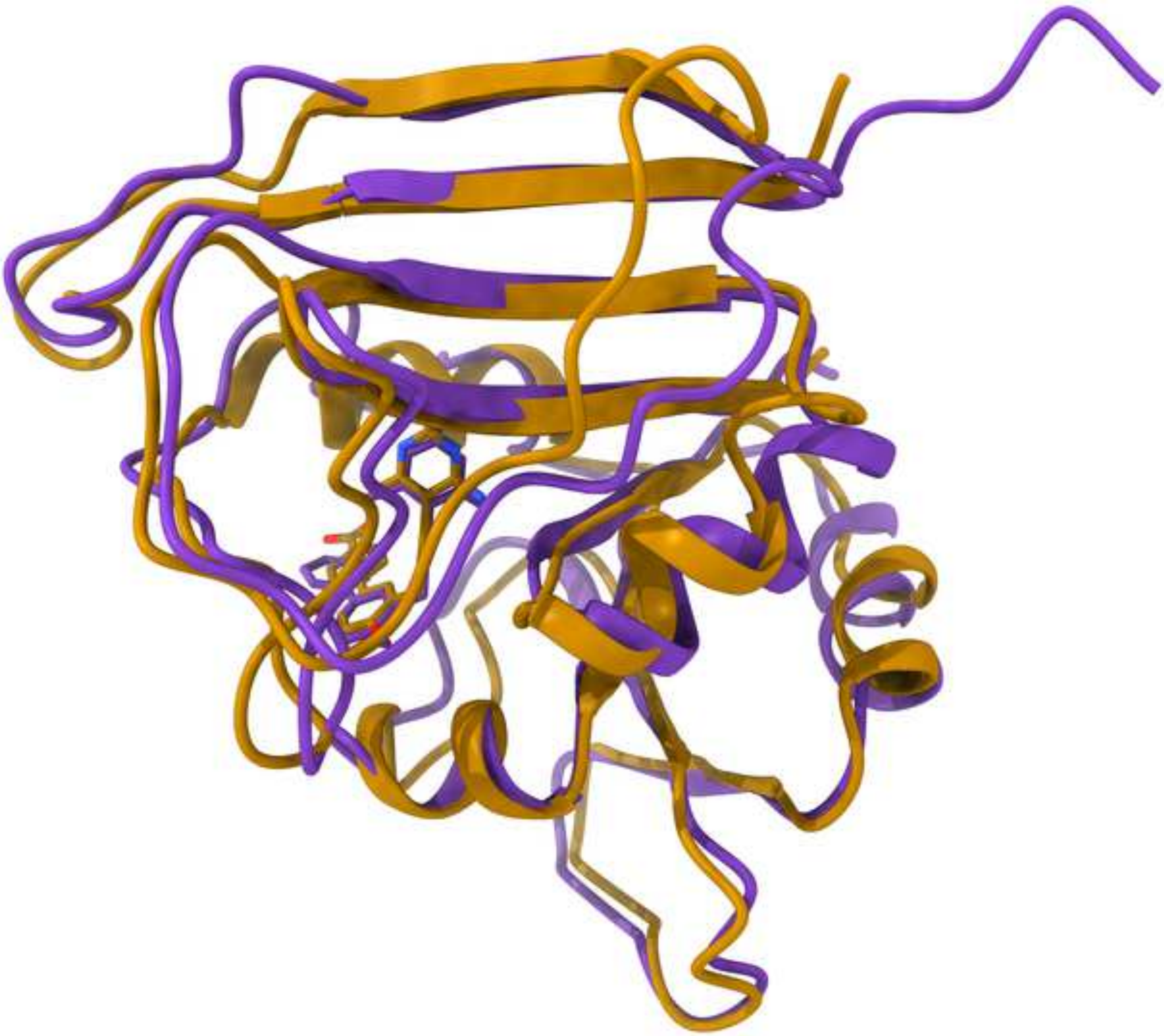

Figure 6

Click here to access/download;Figure;Figure6.tif

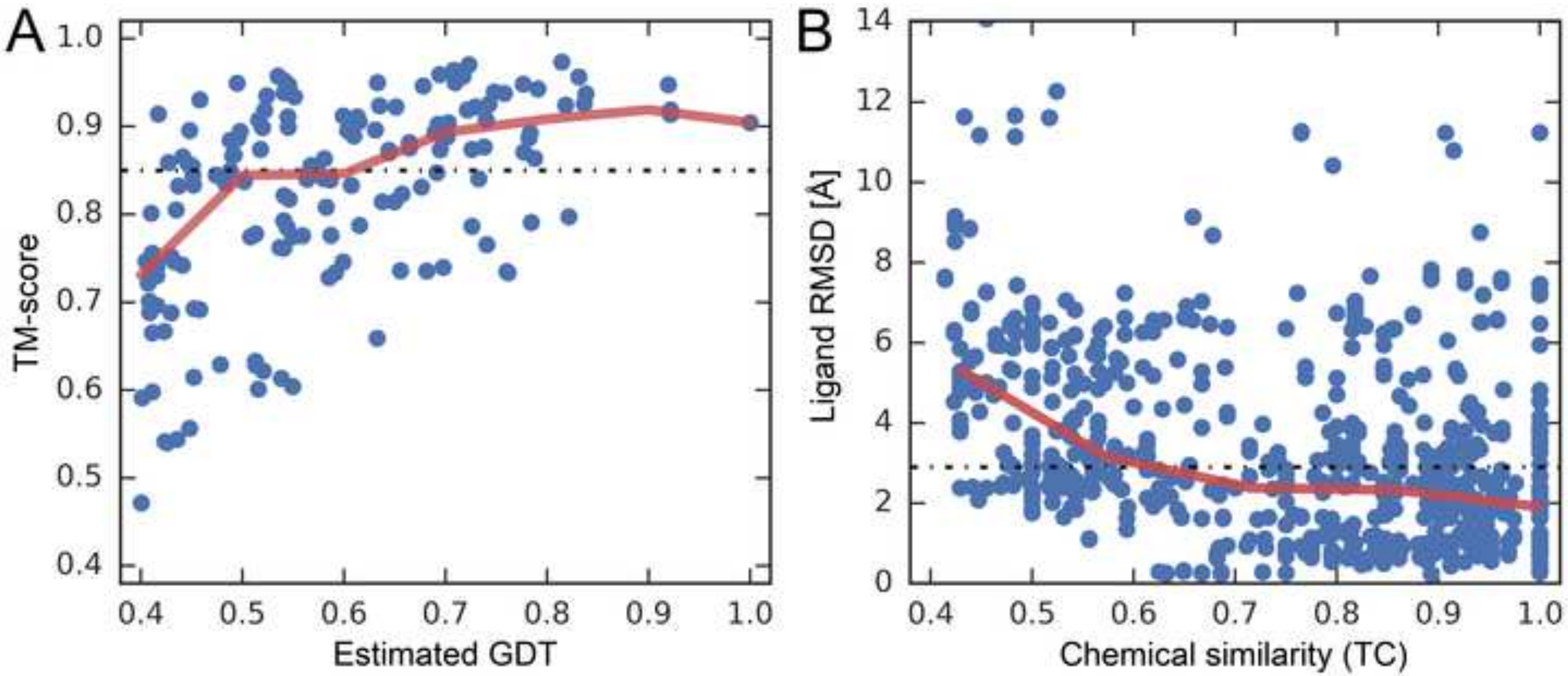

Figure 7

Click here to access/download;Figure;Figure7.tif

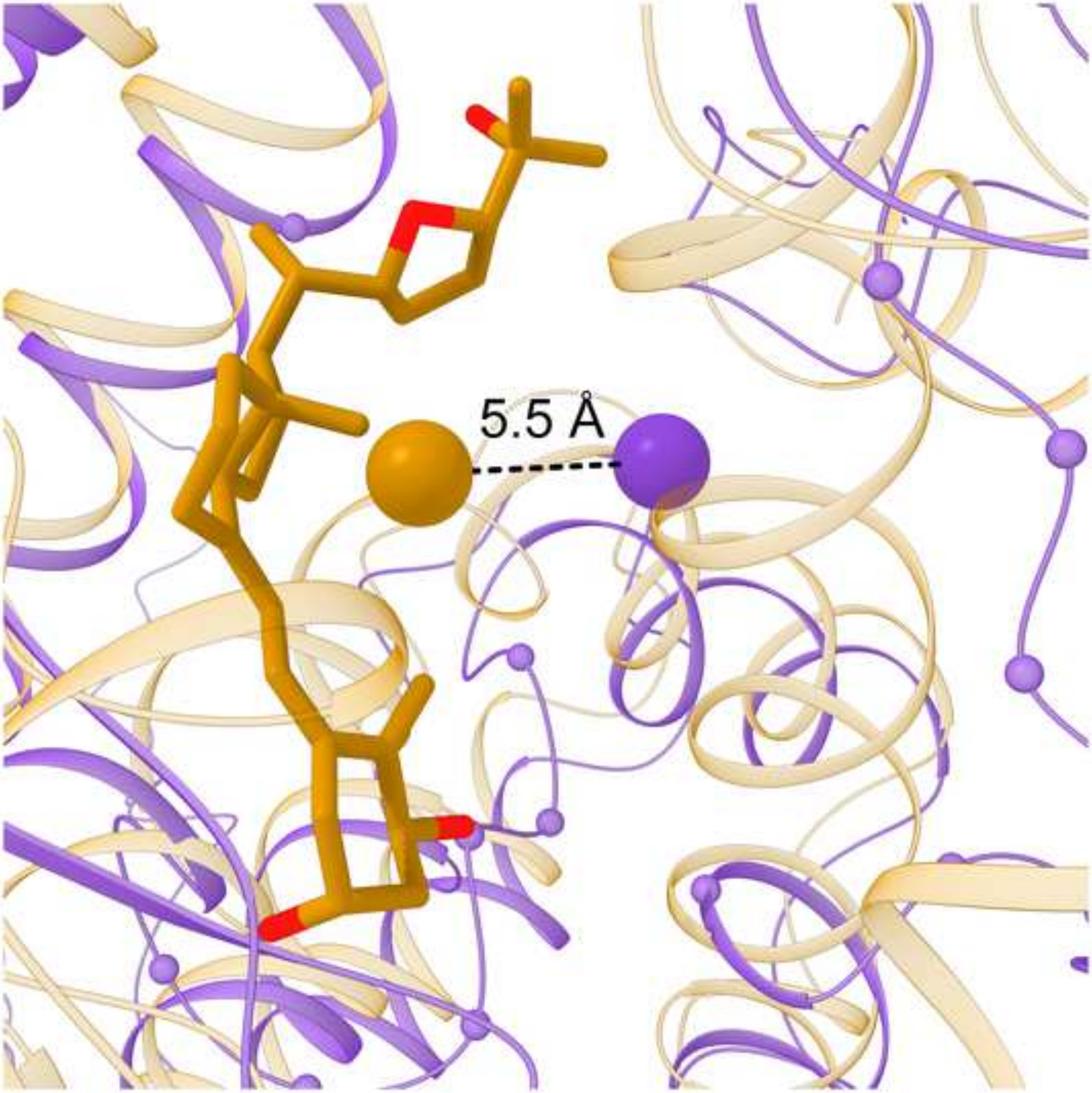

Figure 8

Click here to access/download;Figure;Figure8.tif

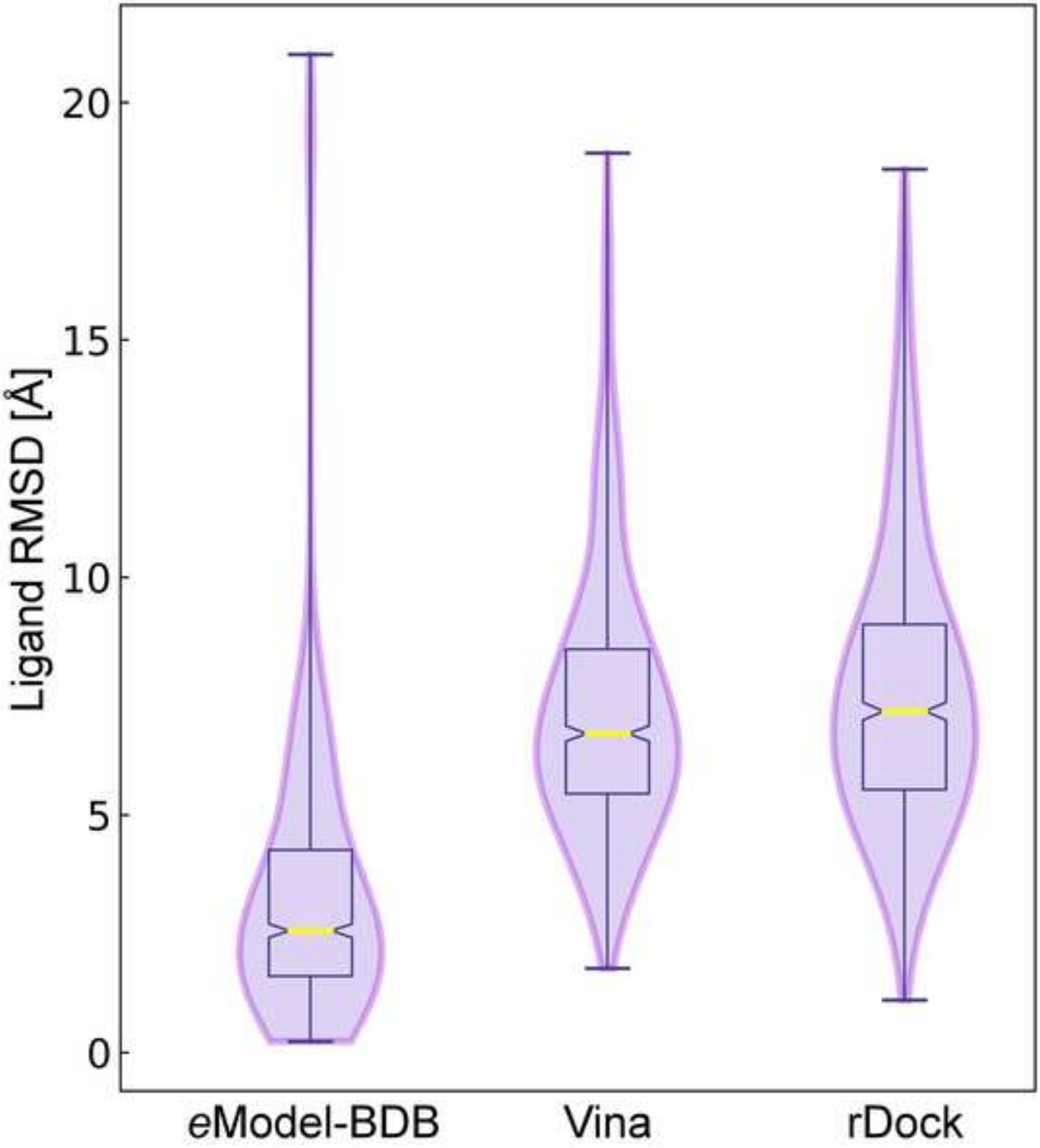

Figure 9

Click here to access/download;Figure;Figure9.tif

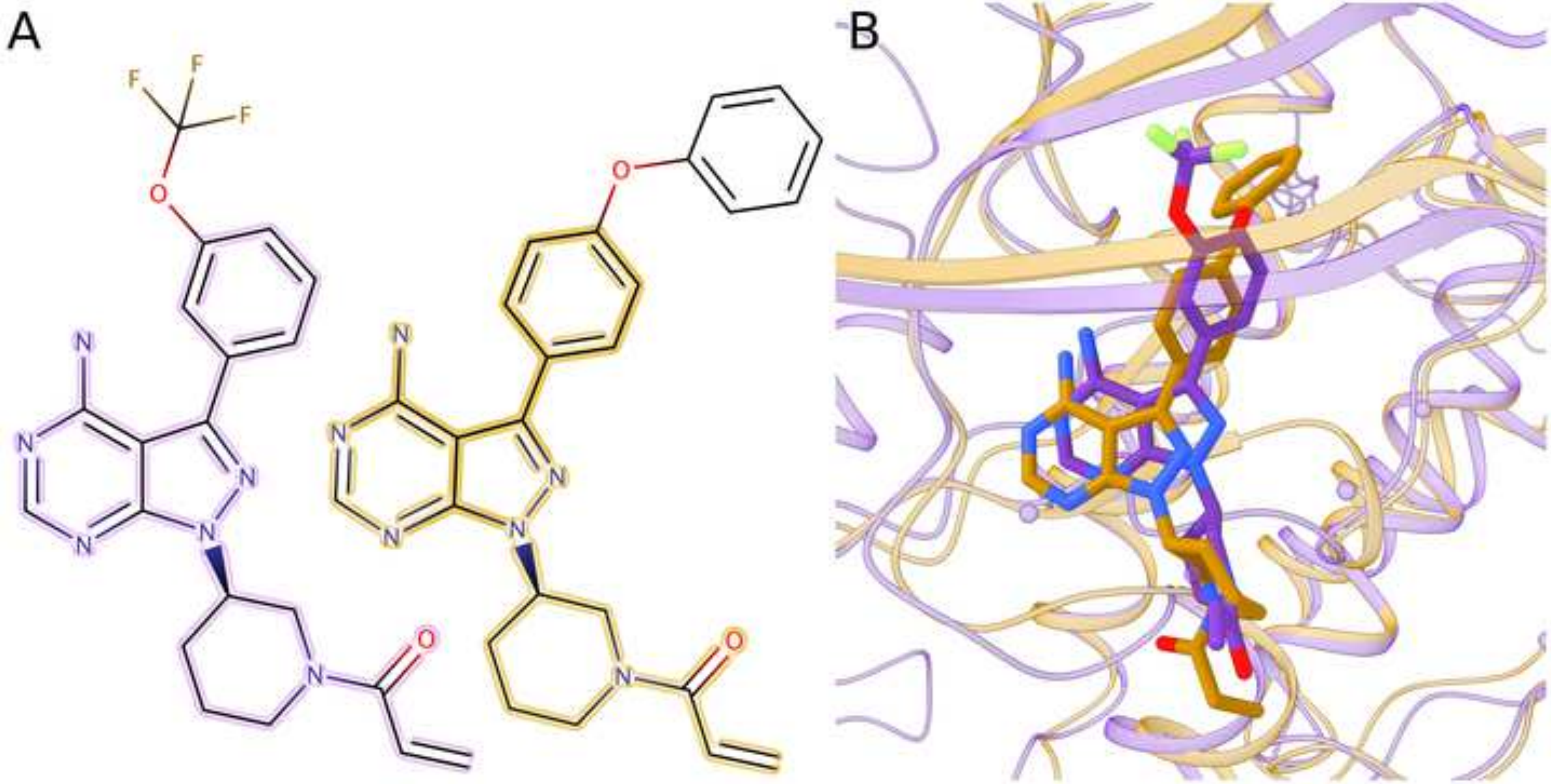

Figure 10 Click here to access/download;Figure;Figure10.tif

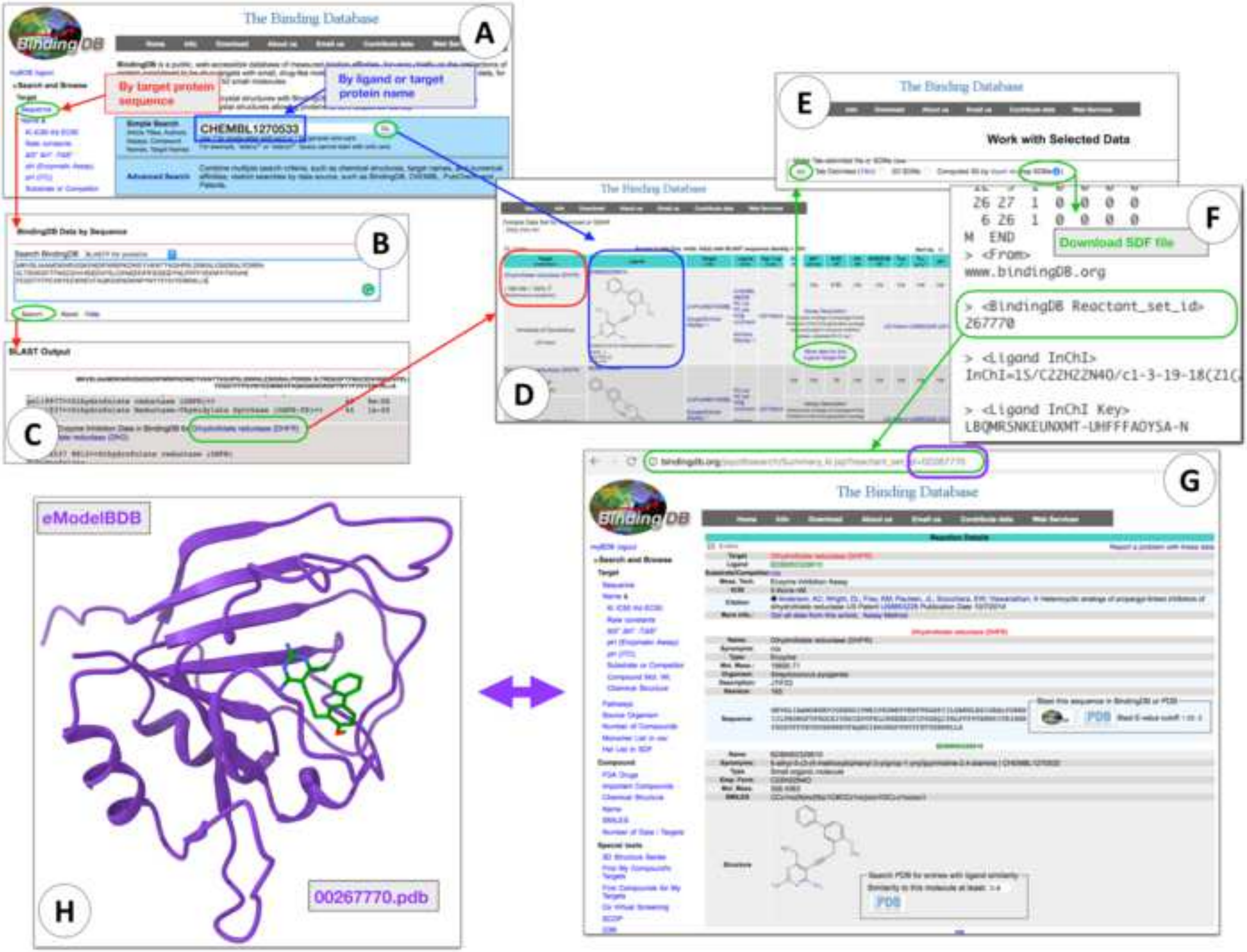

Supplementary File S1

Click here to access/download
Supplementary Material
SupplementaryFileS1.csv

Supplementary File S2

Click here to access/download
Supplementary Material
SupplementaryFileS2.csv

Click here to access/download
Supplementary Material
ResponseLetter.pdf
