# Supplementary material for: eModel-BDB: a database of comparative structure models of drug-target interactions from the Binding Database
Source: Gigascience. 2018 Jul 24;7(8):giy091. doi: 10.1093/gigascience/giy091 (PMC6131211; doi:10.1093/gigascience/giy091)
Supplement: Response_to_Reviewer_Comments_Original_Submission.pdf [file giy091_response_to_reviewer_comments_original_submission.pdf]

Author's Response To Reviewer Comments

Close

Our responses are provided in a separate document "ResponseLetter.pdf" uploaded as Supplementary Material.

Close
