# Supplementary material for: eModel-BDB: a database of comparative structure models of drug-target interactions from the Binding Database
Source: Gigascience. 2018 Jul 24;7(8):giy091. doi: 10.1093/gigascience/giy091 (PMC6131211; doi:10.1093/gigascience/giy091)
Supplement: Reviewer_1_Report_(Original_Submission) -- Janez Konc — 11/22/2017 Reviewed [file giy091_reviewer_1_report_(original_submission).pdf]

Reviewer Report

Title: **eModel-BDB: A database of comparative structure models of drug-target interactions from the Binding Database**

Version: **Original Submission** **Date:** 11/22/2017

Reviewer name: **Janez Konc**

Reviewer Comments to Author:

The authors developed a database of drug-target models that cover the Binding Database that could be used in drug discovery - especially genome-wide virtual screening to support drug repurposing.SPECIFIC COMMENTS1. page 5, lines 1-22: It is not clear how BindingDB drugs are assigned to the predicted pockets with fingerprint-based virtual screening. How is the rank of the drug against pockets detect by eFindSite computed? This should be elaborated.2. page 5, lines 31-32: Why is global structure similarity measure (TM-score) used? To measure the quality of alignment of ligand-bound template binding sites onto the apo-binding site, using some local similarity measure would be better.3. page 7, lines 47-48: The median RMSD of 2.9 Å for the binding pocket (comparison between the generated model and experimental PDB structure) seems rather high. A comment should be added how this could affect drug discovery with docking approaches.4. page 8, lines 23-24: Several other pocket matching algorithms exist and have been used for drug discovery, but the authors provide only two of their own recent works (refs 24 and 25) currently at the submission stage. Consider discussing other successful recent examples as well. GENERAL COMMENTS1. Is the study accounting for biological assemblies, i.e., biologically relevant complexes of proteins? For example, many binding site pockets are between two proteins that form a protein-protein complex. Such pockets are only complete if both interacting protein structures are considered. If this is not the case, this may be a drawback. It should be discussed how biological assemblies were considered.

Level of Interest

Please indicate how interesting you found the manuscript: An article of importance in its field

Quality of Written English

Please indicate the quality of language in the manuscript: Acceptable

Declaration of Competing Interests

Please complete a declaration of competing interests, considering the following questions:

- Have you in the past five years received reimbursements, fees, funding, or salary from an organisation that may in any way gain or lose financially from the publication of this manuscript, either now or in the future?
- Do you hold any stocks or shares in an organisation that may in any way gain or lose financially from the publication of this manuscript, either now or in the future?
- Do you hold or are you currently applying for any patents relating to the content of the manuscript?

- Have you received reimbursements, fees, funding, or salary from an organization that holds or has applied for patents relating to the content of the manuscript?
- Do you have any other financial competing interests?
- Do you have any non-financial competing interests in relation to this paper?

If you can answer no to all of the above, write 'I declare that I have no competing interests' below. If your reply is yes to any, please give details below.

No.

I agree to the open peer review policy of the journal. I understand that my name will be included on my report to the authors and, if the manuscript is accepted for publication, my named report including any attachments I upload will be posted on the website along with the authors' responses. I agree for my report to be made available under an Open Access Creative Commons CC-BY license (http://creativecommons.org/licenses/by/4.0/). I understand that any comments which I do not wish to be included in my named report can be included as confidential comments to the editors, which will not be published.

I agree to the open peer review policy of the journal

To further support our reviewers, we have joined with Publons, where you can gain additional credit to further highlight your hard work (see: https://publons.com/journal/530/gigascience). On publication of this paper, your review will be automatically added to Publons, you can then choose whether or not to claim your Publons credit. I understand this statement. Yes
