# Supplementary material for: eModel-BDB: a database of comparative structure models of drug-target interactions from the Binding Database
Source: Gigascience. 2018 Jul 24;7(8):giy091. doi: 10.1093/gigascience/giy091 (PMC6131211; doi:10.1093/gigascience/giy091)
Supplement: Reviewer_2_Report_(Original_Submission) -- Michael K Gilson — 11/25/2017 Reviewed [file giy091_reviewer_2_report_(original_submission).pdf]

Reviewer Report

Title: **eModel-BDB: A database of comparative structure models of drug-target interactions from the Binding Database**

Version: **Original Submission** **Date:** 11/25/2017

Reviewer name: **Michael K Gilson**

Reviewer Comments to Author:

This manuscript describes the assignment of computed 3D ligand-protein structures to a vast set of ligand-protein interactions for which affinity data are available in the BindingDB database. The project appears to be thoughtfully executed, and is notable for its inclusion of a validation step based on new structures deposited in the PDB subsequent to the initial processing. The structural RMSD values listed at the bottom of page 7 are impressively good. I am confident that many users will find the computed structures provided her to be useful in their own work, as indicated by the authors. I have only a few small suggestions for the paper.Although the title is "A database...", the format of the database is not clear. Will the models be searchable and browse-able, for example? Also, it would be interesting to know if there is a plan to expand the database as more data enter BindingDB.Page 3, line 30, delete "a" before "high-throughput"Page 4, last line: it would be helpful to provide information on how good a GDT-score of >0.4 is. (Perhaps this is in the text and I missed it.)Page 5, line 4: "Confident structure models...": does this mean those models with a GDT-score of >0.4? If not, then how was confidence defined here?

Level of Interest

Please indicate how interesting you found the manuscript: An article of importance in its field

Quality of Written English

Please indicate the quality of language in the manuscript: Acceptable

Declaration of Competing Interests

Please complete a declaration of competing interests, considering the following questions:

- Have you in the past five years received reimbursements, fees, funding, or salary from an organisation that may in any way gain or lose financially from the publication of this manuscript, either now or in the future?
- Do you hold any stocks or shares in an organisation that may in any way gain or lose financially from the publication of this manuscript, either now or in the future?
- Do you hold or are you currently applying for any patents relating to the content of the manuscript?
- Have you received reimbursements, fees, funding, or salary from an organization that holds or has applied for patents relating to the content of the manuscript?
- Do you have any other financial competing interests?
- Do you have any non-financial competing interests in relation to this paper?

If you can answer no to all of the above, write 'I declare that I have no competing interests' below. If your reply is yes to any, please give details below.

The present manuscript describes a project that makes extensive use of the BindingDB database, which my lab developed and maintains.

I agree to the open peer review policy of the journal. I understand that my name will be included on my report to the authors and, if the manuscript is accepted for publication, my named report including any attachments I upload will be posted on the website along with the authors' responses. I agree for my report to be made available under an Open Access Creative Commons CC-BY license (http://creativecommons.org/licenses/by/4.0/). I understand that any comments which I do not wish to be included in my named report can be included as confidential comments to the editors, which will not be published.

I agree to the open peer review policy of the journal

To further support our reviewers, we have joined with Publons, where you can gain additional credit to further highlight your hard work (see: https://publons.com/journal/530/gigascience). On publication of this paper, your review will be automatically added to Publons, you can then choose whether or not to claim your Publons credit. I understand this statement. Yes
