# Supplementary material for: eModel-BDB: a database of comparative structure models of drug-target interactions from the Binding Database
Source: Gigascience. 2018 Jul 24;7(8):giy091. doi: 10.1093/gigascience/giy091 (PMC6131211; doi:10.1093/gigascience/giy091)
Supplement: Reviewer_2_Report_(Revision_1) -- Michael K Gilson — 4/12/2018 Reviewed [file giy091_reviewer_2_report_(revision_1).pdf]

**Reviewer Report**

**Title: eModel-BDB: A database of comparative structure models of drug-target interactions from the Binding Database**

**Version: Revision 1 Date:** 4/12/2018

**Reviewer name: Michael K Gilson**

**Reviewer Comments to Author:**

I havThis paper describes a new database that is likely to be of substantial interest and value to the scientific community. The work appears basically sound, but might benefit from some clarification and enhancements, including in relation to the validation statistics, as now detailed.

The attention to validation of the modeled structures is very welcome, but perhaps a little unclear. For example, text on page 8 says the models were tested against 7,012 experimental structures, but I am not sure what the results of these tests were. The text does says the results were validated for 952 reaction set IDs, but I'm not sure how this relates to the 7,012 cases. What fraction of tests were passed, and what was the definition of passing? Perhaps this is stated, and I missed it.

Also, on page 9, a median distance between predicted and actual pocket locations of 6 A does not sound that good, but the ligand RMSDs reported in Figure 8 seem quite good. It might be helpful to clarify the relationship between these figures of merit. Also, please clarify the dataset plotted in Figure 8. Is this for the 7,012 structures mentioned above?

Finally, regarding the database itself, I think that entries, such as http://www.brylinski.org/pub/eModelBDB.php?reactant_set_id=00267770, would be improved by including the source organism and common name of the protein and possibly its amino acid sequence; a SMILES and/or INCHI string of the compound; and possibly the IDs of the PDB structure(s) used to build the model. This added information would make each entry more self-contained, rather than requiring users to refer back to the PDB and BindingDB for this basic information.e no further comments.

**Methods**

Are the methods appropriate to the aims of the study, are they well described, and are necessary controls included? Yes

**Conclusions**

Are the conclusions adequately supported by the data shown? Yes

**Reporting Standards**

Does the manuscript adhere to the journal's guidelines on minimum standards of reporting? No

Choose an item.

**Statistics**

Are you able to assess all statistics in the manuscript, including the appropriateness of statistical tests used? No, and I do not feel adequately qualified to assess the statistics.

**Quality of Written English**

Please indicate the quality of language in the manuscript: Acceptable

**Declaration of Competing Interests**

Please complete a declaration of competing interests, considering the following questions:

- Have you in the past five years received reimbursements, fees, funding, or salary from an organisation that may in any way gain or lose financially from the publication of this manuscript, either now or in the future?
- Do you hold any stocks or shares in an organisation that may in any way gain or lose financially from the publication of this manuscript, either now or in the future?
- Do you hold or are you currently applying for any patents relating to the content of the manuscript?
- Have you received reimbursements, fees, funding, or salary from an organization that holds or has applied for patents relating to the content of the manuscript?
- Do you have any other financial competing interests?
- Do you have any non-financial competing interests in relation to this paper?

If you can answer no to all of the above, write 'I declare that I have no competing interests' below. If your reply is yes to any, please give details below.

I have no competing interests.

I agree to the open peer review policy of the journal. I understand that my name will be included on my report to the authors and, if the manuscript is accepted for publication, my named report including any attachments I upload will be posted on the website along with the authors' responses. I agree for my report to be made available under an Open Access Creative Commons CC-BY license (http://creativecommons.org/licenses/by/4.0/). I understand that any comments which I do not wish to be included in my named report can be included as confidential comments to the editors, which will not be published.

I agree to the open peer review policy of the journal

To further support our reviewers, we have joined with Publons, where you can gain additional credit to further highlight your hard work (see: https://publons.com/journal/530/gigascience). On publication of this paper, your review will be automatically added to Publons, you can then choose whether or not to claim your Publons credit. I understand this statement.

Yes Choose an item.
