# Supplementary material for: eModel-BDB: a database of comparative structure models of drug-target interactions from the Binding Database
Source: Gigascience. 2018 Jul 24;7(8):giy091. doi: 10.1093/gigascience/giy091 (PMC6131211; doi:10.1093/gigascience/giy091)
Supplement: Reviewer_3_Report_(Original_Submission) -- Takeshi Kawabatag — 12/3/2017 Reviewed [file giy091_reviewer_3_report_(original_submission).pdf]

Reviewer Report

Title: **eModel-BDB: A database of comparative structure models of drug-target interactions from the Binding Database**

Version: **Original Submission** **Date:** 12/3/2017

Reviewer name: **Takeshi Kawabatag**

Reviewer Comments to Author:

This article reports about a database ("eModel-BDB") of 3D models of ligand-bound conformations of drugs and proteins. They claimed that this database contains 200,008 high quality models. As far as I know, this database is the first attempt for collecting the model of ligand-protein complexes, very comprehensively. I appreciate that point. However, this article does not contain any Web address, it just describes " eModel-BDB data will be made freely available through the GigaScience repository". It probably means that the reviewers (including me) cannot access the data during reviewing process, and means that these 200,008 PDB files will be just stored in the GigaDB without any searching services and GUIs. Molecular databases with more than 200,000 entries should have an interface to search data by names of proteins and compounds and by protein sequences and chemical structures. From the users' perspective, these data should be stored in the WEB database with a good searching service, not just in archives; it also should be updated regularly. Of course, the decision completely depends on the editor, because I do not completely understand the policy of the journal Giga Science and GigaDB. I also think that storing in the archive is much better than evaporating, if the authors cannot develop the WEB for some reasons.Even if the editors decide to accept the 200,008 PDB files in the GigaDB, I think this article should describe more about the statistics and examples of their 200,008 structural models to enhance the value of their data and to learn wisdoms from the trial to create huge amount of the models.MAJOR POINTS1) The authors claimed that 2,291 ligand-protein crystal structures with BindingDB affinities are available in PDB, and they made 200,008 drug complex models. Potential users of this database would want to know whether their target proteins and compounds are included in the database or not. Of course, a good searching engine should be available to satisfy users' request, if possible. Instead of that, the authors should prepare the list of protein names (or UniProt ID) or family names (Pfam or SCOP) frequently appeared in their 200,008 models and the 2291 crystal structures. It will be helpful to understand which proteins and families are mostly compensated by their database. If the authors find some biases of ligand types in their models, they also enhance this article. 2) Generally speaking, qualities of comparative models strongly depend on similarities between targets and templates. The authors should show five graphs (1D histogram or 2D histograms) for distribution of the similarities among the 200,008 models. a) sequence identity between the target protein sequence and the template, b) tanimoto coefficient between the target chemical structure and the template, c) TM-score between the target protein model and the template protein model from the ligand-protein complex, d) PMD-distance between the target complex model and the template complex structure, e)2D-histrogram or 2D plots for TM-score and TC-score between the target complex model and the template complex structure.3) The 7,012 experimental structures solved after the modeling are valuable to know which quality scores are correlated with the error. Addition to Figure 2, the authors should add the plot between quality scores (sequence identity, TM-score, Tanimoto coeffieicent, PMD-score, sequence identity) versus TM-score, the pocket distance, and the Ligand RMSD.4) The table containing pairs of the model ID and PDB ID for the 7,012 experimental structures should be provided as Supplementary data. 5) Some figures of 3D models will attract readers. Sets of {template structure, model structure, correct structure} should be shown. I recommend to choose models with median qualities; TM-score is about 0.90, pocket distance is about 5.5 Å, and ligand RMSD is about 2.9 Å. 6) There is no clear description about the version of PDB and Binding DB used for the modeling. The authors wrote "the construction of the structure models has been completed in January 2017", but they should more clearly state the version of the PDB for users, such as 2017/01/04, 2017/01/11, 2017/01/18 or 2017/01/25. The authors should also mention about the updating plan. If they have no plan for updating, they have to write that.

Level of Interest

Please indicate how interesting you found the manuscript: An article of importance in its field

Quality of Written English

Please indicate the quality of language in the manuscript: Acceptable

Declaration of Competing Interests

Please complete a declaration of competing interests, considering the following questions:

- Have you in the past five years received reimbursements, fees, funding, or salary from an organisation that may in any way gain or lose financially from the publication of this manuscript, either now or in the future?
- Do you hold any stocks or shares in an organisation that may in any way gain or lose financially from the publication of this manuscript, either now or in the future?
- Do you hold or are you currently applying for any patents relating to the content of the manuscript?
- Have you received reimbursements, fees, funding, or salary from an organization that holds or has applied for patents relating to the content of the manuscript?
- Do you have any other financial competing interests?
- Do you have any non-financial competing interests in relation to this paper?

If you can answer no to all of the above, write 'I declare that I have no competing interests' below. If your reply is yes to any, please give details below.

I declare that I have no competing interests.

I agree to the open peer review policy of the journal. I understand that my name will be included on my report to the authors and, if the manuscript is accepted for publication, my named report including any attachments I upload will be posted on the website along with the authors' responses. I agree for my report to be made available under an Open Access Creative Commons CC-BY license (http://creativecommons.org/licenses/by/4.0/). I understand that any comments which I do not wish to be included in my named report can be included as confidential comments to the editors, which will not be published.

I agree to the open peer review policy of the journal

To further support our reviewers, we have joined with Publons, where you can gain additional credit to further highlight your hard work (see: https://publons.com/journal/530/gigascience). On publication of this paper, your review will be automatically added to Publons, you can then choose whether or not to claim your Publons credit. I understand this statement. Yes
