# Supplementary material for: eModel-BDB: a database of comparative structure models of drug-target interactions from the Binding Database
Source: Gigascience. 2018 Jul 24;7(8):giy091. doi: 10.1093/gigascience/giy091 (PMC6131211; doi:10.1093/gigascience/giy091)
Supplement: Reviewer_3_Report_(Revision_1) -- Takeshi Kawabatag — 4/17/2018 Reviewed [file giy091_reviewer_3_report_(revision_1).pdf]

Reviewer Report

Title: **eModel-BDB: A database of comparative structure models of drug-target interactions from the Binding Database**

Version: **Revision 1** **Date:** 4/17/2018

Reviewer name: **Takeshi Kawabata**

Reviewer Comments to Author:

I am almost satisfied by added graphs and descriptions which I requested. I also appreciate the Editor to allow me to download the files of the models. However, I still think a database of comparative modelled structures has to be updated regularly using the latest PDB, and has to have the WEB interface for the searching. I will be happy if the authors modify following points before publication.

A MAJOR REQUIREMENT BEFORE PUBLISHING
1) The date of PDB has to be clearly written in the Abstract. Because the 200,005 models stored in the GigaDB is the models generated with the rather old version of PDB (2017/01/31), users should recognize they have to check the latest PDB, before using these models. And total number of Binding DB interactions also has to be described. The ratio of modeled interaction is very valuable for this comprehensive study.

[Abstract]
Results: We created eModel-PDB, a database of 200,005 comparative models of drug-bound proteins based on interaction data obtained from the Binding Database.

=>

Results: We created eModel-PDB, a database of 200,005 comparative models of drug-bound proteins based on 1,391,403 interaction data obtained from the Binding Database, with the PDB library of January 31, 2017.

MINOR REQUIREMENTS
1) Page 8, line 49: 5isp, chain A, => 5isp, chain X,
2) The reactant set ID of the structure in Fig. 7 has to be added.
3) I did not understand the modeling and evaluating procedure described in the "Ligand docking" section (page 7) and Fig. 9, for the reactant ID 50103430 (CYP17A1 with BDBM50061174). The authors used three structures for templates and evaluations:
Monomer template: 1z11_C (seqid=29.5%) with ligand "8MO" (TC=0.41)
Complex template: 3ruk_D (seqid=68.2%) with ligand "AER" (TC=0.89)
Correct complex: 5irq_D(seqid=64.0%) with ligand "7D6" (TC=0.54).

Among the three structures, 3ruk is the most similar structure both for the protein and the ligand. I have two questions. 1) Why the authors used 1z11 to model the monomeric structure, instead of 3ruk ? 2) Why the authors used 5irq to validate their model ? The template 3ruk is more similar than 5irq. Please answer these two questions. I think more suitable example than this should be shown.

Methods

Are the methods appropriate to the aims of the study, are they well described, and are necessary controls included? Choose an item.

Conclusions

Are the conclusions adequately supported by the data shown? Choose an item.

Reporting Standards

Does the manuscript adhere to the journal's guidelines on minimum standards of reporting? Choose an item.

Choose an item.

Statistics

Are you able to assess all statistics in the manuscript, including the appropriateness of statistical tests used? No, and I do not feel adequately qualified to assess the statistics.

Quality of Written English

Please indicate the quality of language in the manuscript: Acceptable

Declaration of Competing Interests

Please complete a declaration of competing interests, considering the following questions:

- Have you in the past five years received reimbursements, fees, funding, or salary from an organisation that may in any way gain or lose financially from the publication of this manuscript, either now or in the future?
- Do you hold any stocks or shares in an organisation that may in any way gain or lose financially from the publication of this manuscript, either now or in the future?
- Do you hold or are you currently applying for any patents relating to the content of the manuscript?
- Have you received reimbursements, fees, funding, or salary from an organization that holds or has applied for patents relating to the content of the manuscript?
- Do you have any other financial competing interests?
- Do you have any non-financial competing interests in relation to this paper?

If you can answer no to all of the above, write 'I declare that I have no competing interests' below. If your reply is yes to any, please give details below.

I declare that I have no competing interests.

I agree to the open peer review policy of the journal. I understand that my name will be included on my report to the authors and, if the manuscript is accepted for publication, my named report including any attachments I upload will be posted on the website along with the authors' responses. I agree for my report to be made available under an Open Access Creative Commons CC-BY license (http://creativecommons.org/licenses/by/4.0/). I understand that any comments which I do not wish to

be included in my named report can be included as confidential comments to the editors, which will not be published.

I agree to the open peer review policy of the journal

To further support our reviewers, we have joined with Publons, where you can gain additional credit to further highlight your hard work (see: https://publons.com/journal/530/gigascience). On publication of this paper, your review will be automatically added to Publons, you can then choose whether or not to claim your Publons credit. I understand this statement.

Yes Choose an item.
