# Supplementary material for: eModel-BDB: a database of comparative structure models of drug-target interactions from the Binding Database
Source: Gigascience. 2018 Jul 24;7(8):giy091. doi: 10.1093/gigascience/giy091 (PMC6131211; doi:10.1093/gigascience/giy091)
Supplement: Supplemental Files [file ResponseLetter.pdf › ResponseLetter.pdf]

Michal Brylinski, Ph.D., Associate Professor
Department of Biological Sciences
Center for Computation & Technology
Louisiana State University
407 Choppin Hall, Baton Rouge, LA 70803
michal@brylinski.org

Sunday, June 03, 2018

Scott Edmunds, Ph.D.
Executive Editor, GigaScience

Dear Dr. Edmunds:

Enclosed please find the second revised version of a manuscript **GIGA-D-17-00258** by M. Naderi, R. Govindaraj, and M. Brylinski entitled "*e*Model-BDB: A database of comparative structure models of drug-target interactions from the Binding Database" submitted for consideration for publication in *GigaScience*.

All authors would like to thank the Editor and Reviewers for the thoughtful reviews of our manuscript. We appreciate their insightful comments on how to improve the paper. Below please find point-by-point responses with relevant parts of the paper marked by red font.

Revisions in response to Reviewer 2:

(1) *The attention to validation of the modeled structures is very welcome, but perhaps a little unclear. For example, text on page 8 says the models were tested against 7,012 experimental structures, but I am not sure what the results of these tests were. The text does says the results were validated for 952 reaction set IDs, but I'm not sure how this relates to the 7,012 cases. What fraction of tests were passed, and what was the definition of passing? Perhaps this is stated, and I missed it.*

7,012 is the total number of experimental structures deposited to PDB after February 2017. The validation set was selected from these 7,012 based on the criteria stated in the same paragraph. Following this Reviewer's suggestion, we added the following clarifications to this section:

Analyses

Page 8 (top), *Data validation*

In addition to the rigorous quality control maintained during dataset generation, *e*Model-BDB is validated retrospectively against experimental structures recently deposited to the PDB. The structure models of BindingDB interactions have been constructed with the PDB library as of January 31st 2017, therefore, to validate *e*Model-BDB models, 7,012 experimental structures deposited to the PDB after February 2017 were considered. The validation protocol is made more challenging by including only remote homology models with a template-target sequence identity of <40%. In order to maximize the validation coverage, we use the recently determined structures of *e*Model-BDB targets and their homologs with at least 40% sequence identity. After applying these filters, 41 recently solved experimental structures selected from the PDB can be used to validate 161 *e*Thread models and 952 BindingDB reaction set IDs, comprising 39 target proteins, 52 pockets, and 881 compounds. This set is referred to as the validation dataset. The list of validation pairs is given in Supplementary File S2.

***(2)** Also, on page 9, a median distance between predicted and actual pocket locations of 6 A does not sound that good, but the ligand RMSDs reported in Figure 8 seem quite good. It might be helpful to clarify the relationship between these figures of merit. Also, please clarify the dataset plotted in Figure 8. Is this for the 7,012 structures mentioned above?*

The pocket distance analysis was conducted for the entire validation dataset of 52 models, whereas the ligand RMSD was calculated for a subset of correctly identified pockets. This is because it does not make sense to report the accuracy of ligand docking for incorrectly detected binding sites. Specifically, the ligand RMSD was calculated only for 37 models whose pocket centers are predicted within 8 Å. We note that the average pocket distance is 4.1 Å for this subset. In response to this comment, we added the following revisions:

<u>Analyses</u>

Page 9 (bottom), *Ligand docking*

Here, we employ a subset of 37 models selected from the validation set whose pocket centers are predicted within 8 Å.

***(3)** Finally, regarding the database itself, I think that entries, such as http://www.brylinski.org/pub/eModelBDB.php?reactant_set_id=00267770, would be improved by including the source organism and common name of the protein and possibly its amino acid sequence; a SMILES and/or INCHI string of the compound; and possibly the IDs of the PDB structure(s) used to build the model. This added information would make each entry more self-contained, rather than requiring users to refer back to the PDB and BindingDB for this basic information.*

To address this Reviewer's comment, we added all requested information to the *e*Model-BDB files, including the source organism, the common name of the protein and its amino acid sequence, and SMILES and INCHI data. The IDs of the PDB structure(s) used to build the initial model, and the template protein and ligand IDs used in similarity-based docking are also included.

Revisions in response to Reviewer 3:

(1) *The date of PDB has to be clearly written in the Abstract. Because the 200,005 models stored in the GigaDB is the models generated with the rather old version of PDB (2017/01/31), users should recognize they have to check the latest PDB, before using these models. And total number of Binding DB interactions also has to be described. The ratio of modeled interaction is very valuable for this comprehensive study. [Abstract] Results: We created eModel-PDB, a database of 200,005 comparative models of drug-bound proteins based on interaction data obtained from the Binding Database. => Results: We created eModel-PDB, a database of 200,005 comparative models of drug-bound proteins based on 1,391,403 interaction data obtained from the Binding Database, with the PDB library of January 31, 2017.*

Abstract in the revised manuscript has been modified exactly as suggested by the Reviewer:

> ***Results.*** We created *e*Model-BDB, a database of 200,005 comparative models of drug-bound proteins based on 1,391,403 interaction data obtained from the Binding Database and the PDB library of January 31, 2017.

(2) *Page 8, line 49: 5isp, chain A, => 5isp, chain X.*

This mistake has been corrected and page 8 (bottom) of the revised manuscript reads:

> This model is validated against the crystal structure of DHFR-UCP1106 from *Staphylococcus aureus* (PDB-ID: 5isp, chain X, 43% sequence identity to the target) released on 2017-06-28 [27].

(3) *The reactant set ID of the structure in Fig. 7 has to be added.*

Following this Reviewer's suggestion, we added the reactant set ID to the manuscript. Page 9 (middle) of the revised manuscript reads:

> This model was constructed for the BindingDB reactant set ID 50662356 based on human retinoic acid receptor RXR-alpha (PDB-ID: 4nqa, chain H, 38% sequence identity to the target) [28].

(4) *I did not understand the modeling and evaluating procedure described in the "Ligand docking" section (page 7) and Fig. 9, for the reactant ID 50103430 (CYP17A1 with BDBM50061174). The authors used three structures for templates and evaluations:*
Monomer template: 1z11_C (seqid=29.5%) with ligand "8MO" (TC=0.41)
Complex template: 3ruk_D (seqid=68.2%) with ligand "AER" (TC=0.89)
Correct complex: 5irq_D(seqid=64.0%) with ligand "7D6" (TC=0.54).
Among the three structures, 3ruk is the most similar structure both for the protein and the ligand. I have two questions. 1) Why the authors used 1z11 to model the monomeric structure, instead of 3ruk ? 2) Why the authors used 5irq to validate their model ? The template 3ruk is

more similar than 5irq. Please answer these two questions. I think more suitable example than this should be shown.

We agree with the Reviewer that this case study may be confusing. Therefore, it is replaced with another example in the revised manuscript. In response to this comment, we added the following revisions:

Analyses

Pages 9 (bottom) and 10, *Ligand docking*

> The model of the BindingDB reactant set ID 50974033 consisting of tyrosine-protein kinase (TSK) complexed with BDBM50399512 is selected to exemplify the accuracy of complex structures in *e*Model-BDB. The model of TSK built on the crystal structure of human hemopoietic cell kinase (HCK) (PDB-ID: 1qcf, chain A, 39% sequence identity to the target) [30] by *e*Thread is assigned an estimated GDT-score of 0.61. Subsequently, the complex model of TSK-BDBM50399512 was constructed by similarity-based docking employing the crystal structure of HCK bound to a pyrazolo-pyrimidine inhibitor (PDB-ID: 3vs7, chain B, 37% sequence identity to the target) [31]. This HCK complex was selected as the best ligand-bound template based on the high TM-score of 0.64 and TC of 0.53, yielding the shortest PMD of 0.35. Figure 9 shows the validation of the modeled TSK-BDBM50399512 by the experimental structure of Bruton's tyrosine kinase (Btk) bound to an anti-cancer drug, ibrutinib (PDB-ID: 5p9i, chain A, TM-score: 0.92, 58% sequence identity to the target) released on 2017-05-24 [32]. Kcombu reports a significant chemical alignment between BDBM50399512 and ibrutinib with a TC of 0.68 (Figure 9A). Upon the superposition of TSK and Btk proteins, the RMSD between BDBM50399512 docked to the model and ibrutinib bound in the experimental structure calculated over the chemical alignment reported by kcombu is 2.62 Å (Figure 9B). These results verify that the computer-generated TSK-BDBM50399512 model for the BindingDB reactant set ID 50974033 is correct.

Figures

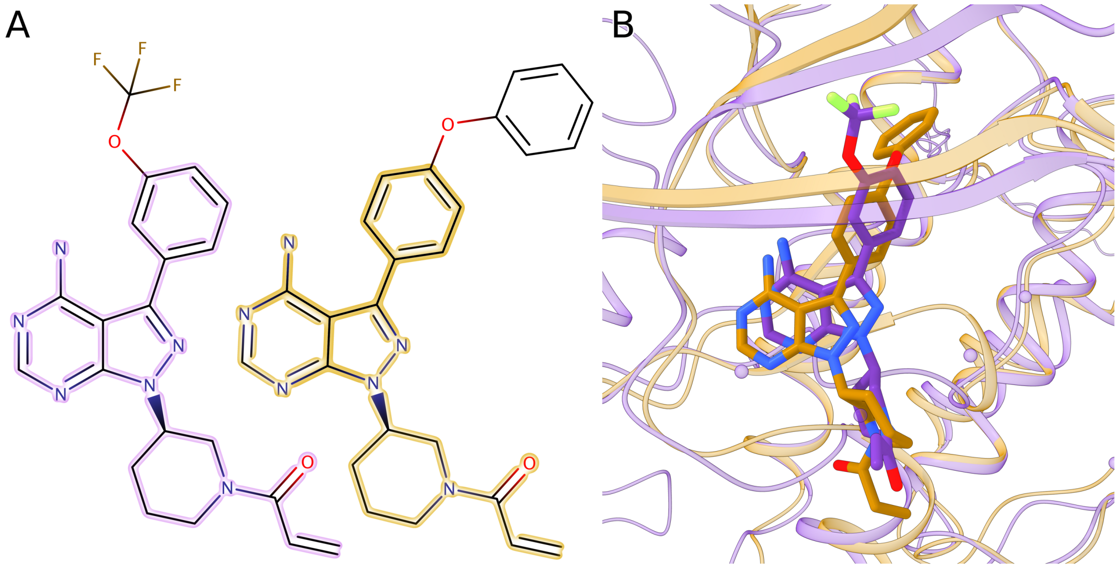

Figure 9. Representative example of a complex structure constructed by similarity-based docking. (**A**) Chemical alignment between BDBM50399512 (left) and ibrutinib (right) reported by kcombu. 26 equivalent atom pairs constituting the maximum common substructure are outlined in purple in BDBM50399512 and in gold in ibrutinib. (**B**) The model of tyrosine-protein kinase (TSK, purple ribbons) is superposed onto the crystal structure of HCK (gold ribbons) complexed with ibrutinib (gold sticks). Cα atoms of binding residues identified in the TSK model by *e*FindSite are shown as purple spheres, whereas the target compound, BDBM50399512, docked into the predicted pocket is represented by purple sticks. Non-carbon atoms in BDBM50399512 and ibrutinib are colored by atom type (O – red, N – blue, F – green).

References

30. Schindler T, Sicheri F, Pico A, Gazit A, Levitzki A and Kuriyan J. Crystal structure of Hck in complex with a Src family-selective tyrosine kinase inhibitor. Mol Cell. 1999;3 5:639-48.
31. Saito Y, Yuki H, Kuratani M, Hashizume Y, Takagi S, Honma T, et al. A pyrrolo-pyrimidine derivative targets human primary AML stem cells in vivo. Sci Transl Med. 2013;5 181:181ra52. doi:10.1126/scitranslmed.3004387.
32. Bender AT, Gardberg A, Pereira A, Johnson T, Wu Y, Grenningloh R, et al. Ability of Bruton's Tyrosine Kinase Inhibitors to Sequester Y551 and Prevent Phosphorylation Determines Potency for Inhibition of Fc Receptor but not B-Cell Receptor Signaling. Mol Pharmacol. 2017;91 3:208-19. doi:10.1124/mol.116.107037.

I hope that you and the Reviewers find the changes satisfactory. Thank you for your consideration of the second revised version of our manuscript.

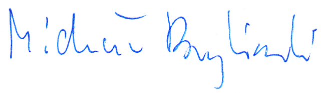

Michal Brylinski
Computational Systems Biology Group @ LSU
